# SMCVdb: a database of experimental cellular toxicity information for drug candidate molecules

**DOI:** 10.1093/database/baae100

**Published:** 2024-10-18

**Authors:** Abhay Deep Pandey, Ghanshyam Sharma, Anshula Sharma, Sudhanshu Vrati, Deepak T Nair

**Affiliations:** Regional Centre for Biotechnology, NCR Biotech Science Cluster, 3rd Milestone, Faridabad-Gurgaon Expressway, Faridabad 121001, India; Regional Centre for Biotechnology, NCR Biotech Science Cluster, 3rd Milestone, Faridabad-Gurgaon Expressway, Faridabad 121001, India; Regional Centre for Biotechnology, NCR Biotech Science Cluster, 3rd Milestone, Faridabad-Gurgaon Expressway, Faridabad 121001, India; Regional Centre for Biotechnology, NCR Biotech Science Cluster, 3rd Milestone, Faridabad-Gurgaon Expressway, Faridabad 121001, India; Regional Centre for Biotechnology, NCR Biotech Science Cluster, 3rd Milestone, Faridabad-Gurgaon Expressway, Faridabad 121001, India

## Abstract

Many drug discovery exercises fail because small molecules that are effective inhibitors of target proteins exhibit high cellular toxicity. Early and effective assessment of toxicity and pharmacokinetics is essential to accelerate the drug discovery process. Conventional methods for toxicity profiling, including *in vitro* and *in vivo* assays, are laborious and resource-intensive. In response, we introduce the Small Molecule Cell Viability Database (SMCVdb), a comprehensive resource containing toxicity data for over 24 000 compounds obtained through high-content imaging (HCI). SMCVdb seamlessly integrates chemical descriptions and molecular weight data, offering researchers a holistic platform for toxicity data aiding compound prioritization and selection based on biological and economic considerations. Data collection for SMCVdb involved a systematic approach combining HCI toxicity profiling with chemical information and quality control measures ensured data accuracy and consistency. The user-friendly web interface of SMCVdb provides multiple search and filter options, allowing users to query the database based on compound name, molecular weight range, or viability percentage. SMCVdb empowers users to access toxicity profiles, molecular weights, compound names, and chemical descriptions, facilitating the exploration of relationships between compound properties and their effects on cell viability. In summary, the database provides experimentally derived cellular toxicity information for over 24 000 drug candidate molecules to academic researchers, and pharmaceutical companies. The SMCVdb will keep growing and will prove to be a pivotal resource to expedite research in drug discovery and compound evaluation.

**Database URL**: http://smcvdb.rcb.ac.in:4321/

## Introduction

The drug discovery process is a complex and challenging task, characterized by high cost, long time, and inherent uncertainty, and nearly 80%–90% of drug developments are abandoned [1–4] even before reaching the level of human testing. Furthermore, 95% of drugs that enter human trials fail to achieve their desired results [5, 6]. Many programs in the early phases of drug discovery focus on identifying compounds that interact with specific targets [7]. Although potency is of critical importance in these early stages, the candidate drug’s therapeutic efficacy and success largely depend on its pharmacokinetic properties and toxicity profile [8]. According to Van Norman’s estimates [9], it is suggested that success rates in clinical trials could see a substantial improvement of about 44% if measures were implemented to minimize or minimize toxicity-related failures.

In recent years, there has been considerable interest in developing new methods for toxicity profiling [10]. Toxicity profiling is a method of assessing the toxicity of compounds [11]. This can be done using a variety of techniques, including *in vitro, in vivo*, and epidemiological studies. *In vivo* and *in vitro* screening techniques are generally helpful in identifying toxicities during different phases of drug development [12, 13].

High-content imaging (HCI) is a powerful technique that can be used to rapidly and quantitatively assess the toxic effects of drugs on a population of cells. HCI uses automated microscopy to image cells and collects data at various levels, such as on cell morphology, cell survival, and expression [14, 15]. Although it takes time to standardize the corresponding cellular assay, HCI can provide quantitative information regarding the property under scrutiny with high throughput. The quantitative information gained from HCI can be used to further develop robust computational approaches for pre-screening and prioritization of potential compounds [16, 17], especially those identified through high-throughput testing [18, 19].

A Database of Small Molecule Cell Viability generated using HCI will be helpful for the researchers to choose small molecules depending on the score of cell viability. This may cut down the time and steps toward the identification of small molecules of desired therapeutic value. However, to the best of our knowledge, there is no such open-access dataset or webserver currently available. Therefore, we aim to develop a database of Small Molecule Cell Viability based on the actual data obtained in HTS. We present the initial version of the database that provides quantitative information regarding the toxicity of more than 24 000 compounds recorded from BHK21 cells using HCI.

## Methods and data collection

### Data collection

A systematic approach was used to obtain and integrate data from different experimental sources to create the Small Molecule Cell Viability Database (SMCVdb). A customized library of 24 361 compounds was procured from ChemBridge corporation. The library exhibits structural diversity, characterized by a broad range of scaffolds and a high abundance of unique pharmacophores. The primary objective was to gather comprehensive toxicity profiling information using HCI for a diverse group of 24 631 compounds. BHK21 cells were seeded at 2000 cells/well in 60 μl of Minimum Essential Medium Eagle in a Corning 384-well black plate. The next day, the drug was diluted in Dulbecco′s Modified Eagle′s Medium supplemented with 2% Fetal bovine serum to a final concentration of 10 μM. Five microliters of the diluted drug were added to each well, and each plate included four columns of Dimethyl sulfoxide as controls. After 22–24 h, the cells were stained with nuclear dyes, including five μg/ml Hoechst and SYTOX Orange, and imaged after 15 min.

HCI images were acquired using the ImageXpress Micro Confocal High-Content Imaging System (Molecular Devices). Four images were captured at a magnification of 10× for each well, using two channels for 4’,6-diamidino-2-phenylindole and Texas Red fluorescence. The acquired images were then analyzed using a custom image analysis program, which relied on nuclear staining to determine the total cell numbers. The resulting data was exported to Microsoft Excel for further analysis and visualization, enabling the comparison of average cell numbers across different treatments involving various molecules. A viability score of 100% indicates no effect compared to the control, while values above 100% suggest increased cell viability, and values below 100% indicate decreased viability. Viability is inversely proportional to toxicity. A higher viability score indicates lower toxicity, whereas a lower viability score suggests higher toxicity. The observed range reflects the variability in the response of BHK21 cells to the tested compounds.

The formula used to calculate the viability score is as follows.


$${\mathrm{No}}{\mathrm{. of\ Viable\ cells}}\;{\mathrm{ = Total\ no}}{\mathrm{. of cells - No}}{\mathrm{. of\ dead\ cells}}{\mathrm{.}}\\[-5pt]$$

$${\mathrm{\%\ Cell\ Viability}}\;{\mathrm{ = }}\left( {{\mathrm{Viability\ of\ sample/Viability\ of\ control}}} \right) \\{\mathrm{ x\ 100}}$$

Additional data for each compound were collected in parallel with the HCI experiments. This included chemical information such as compound identification (ID), molecular weight, and compound name. The compound “SMCV ID” is a unique identifier within the SMCVdb and is hyperlinked to the ChemBridge website. Similarly, the PubChem CID also links to the corresponding entry in PubChem. This connectivity allows researchers to access comprehensive chemical descriptions, structural information, and pricing options for the corresponding compound. Data from HCI experiments, along with associated chemical information, were compiled and organized in a structured format suitable for database integration. Quality control checks were implemented to ensure accuracy and consistency throughout the dataset.

### Database creation

The user-friendly SMCVdb dashboard was developed in R (v3.6.3) using Shiny (v1.8.0) [20, 21]. The data compilation process involved creating a CSV file containing metadata that includes SMILES notations, viabilities, and reference images for small molecules. These data were seamlessly integrated into an SQLite database using RSQLite (v2.2.14), ensuring efficient storage and retrieval within the Shiny application [22]. Additionally, libraries such as progress (v1.2.2) were employed to merge images, and EBImage (v4.28.1) was utilized to add color and brightness to enhance the visual representation [23, 24]. The Shiny framework enables a dynamic user interface with interactive data visualization and user engagement. We leverage the DT package (v0.23) to implement interactive data tables, providing features like sorting, filtering, and pagination for efficient data exploration [25]. To enhance the visual representation of reference images, a custom function utilizes Formattable (v0.2.1) to display images in pop-up windows when corresponding entries are clicked [26]. The user interface is structured into tabs for different functionalities using the shiny dashboard package (v0.7.2), while packages like Dplyr (1.0.10), Evaluate (0.19), and Shiny Widgets (1.0.1) contribute to enhanced functionality and user experience [27–29]. The SQLite database ensures swift and reliable data access, minimizing loading times and facilitating seamless exploration. The combination of dynamic data visualization, interactive tables, and image pop-ups provides researchers with an engaging and informative platform for identifying patterns and trends within the data. The user-centric design prioritizes intuitive navigation and clear instructions, ensuring effective interaction with data and visualizations. Moreover, the flexibility of R and Shiny allows for easy customization and extension of the dashboard’s functionalities to meet specific research needs.

## Results

### Small molecule cell viability database

To set up the Small Molecule Cell Viability Database, we used HCI to evaluate the toxicity of the compounds against the BHK21 cell line. Our findings revealed considerable variability in toxicity compounds, with some compounds exhibiting significant toxicity while others exhibited minimal side effects. The SMCVdb is a valuable resource for researchers interested in analyzing the toxicity of chemicals. It facilitates the identification of potentially toxic compounds and the assessment of toxicity of existing compounds. By combining toxicity data obtained through HCI with comprehensive chemical descriptions and pricing information from the source vendor, the SMCVdb provides a central and accessible platform for investigating relationships between small molecule formulations between materials and cell membranes.

The creation of the SMCVdb involved a systematic approach involving the integration of data from different studies. We used cellular models and assays to fully investigate the effects of the compound on cell viability and experimental conditions. Using multi-core imaging technologies, we captured detailed cellular data and simultaneously analyzed multiple parameters, including morphology, quantification, and specificity of biomarker expression. Each entry in the SMCVdb is assigned a unique SMCV ID, which provides direct navigation and access to specific features of interest. “Source ID” links to the source website, providing researchers direct access to search and purchase detailed chemical information, structural descriptions, and pricing options. These chemical descriptions and feasibility data are integrated into the database and enable researchers to make informed decisions on drug selection and prioritization considering biological and cost considerations. The database includes molecular weight data, an important physicochemical property known to affect pharmacokinetics, availability, and potential toxicological effects, by enabling researchers to explore the relationship between compound size and feasibility profile. The database facilitates the identification of compounds with size-based toxicity and helps explore structure–activity relationships that contribute to the development of smaller drugs that are safe and effective.

### SMCVdb features and functionality

**Figure 1.**
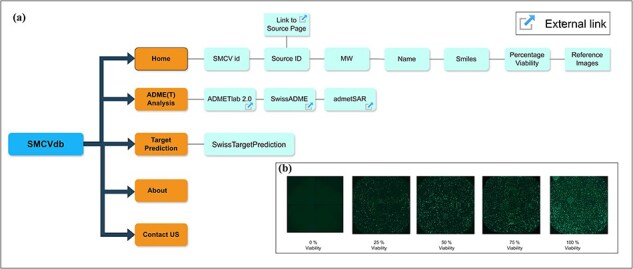
Information and relationships of entities in SMCVdb: (a) Relationships between the entities in SMCVdb is shown with boxes and lines referring to the information and functions in SMCVdb and (b) Sample images showing percentage viability at different viability levels (e.g.: 0, 25, 50, 75, and 100).

With its user-friendly interface, SMCVdb allows researchers to effortlessly search, filter, and visualize data. There is a centralized search option where users can search using keywords and based on specific criteria such as drug name, molecular weight, and percentage viability, which allows searching for compounds with desired properties or target toxicity information. Detailed information about the relationships of entities of SMCVdb is shown in Fig. 1a. For reference to viability percentage data, users can refer to Fig. 1b. Users can sort and filter the numeric entries as ID, molecular weight, and viability in an increasing or decreasing manner. The use of the SMCVdb research database is straightforward and widespread, proving to be a valuable resource for pharmaceutical companies, contract research organizations, and regulatory agencies involved in drug discovery and drug safety research. We also provide links to the servers for additional ADME(T) analysis and target search. Links are provided to obtain detailed chemical and pricing information regarding each small molecule. A link is also provided to download the entire data table without images. The integration of toxicity, chemical, and pricing information into the database will considerably hasten the decision-making process regarding lead selection, cost-effective purchasing, and lead optimization. In summary, the SMCVdb serves as a comprehensive resource that seamlessly integrates cell viability data acquired through 2D imaging with comprehensive drug descriptions and pricing details for 24 361 compounds. Additionally, it encompasses essential pharmacokinetic information, chemical data, and vendor source links. The SMCVdb entries functionality is further enhanced by linking it with associated PubChem CIDs through a dedicated column [30]. The linking allows seamless access to the corresponding entries in the PubChem database. Also, the compounds with PubChem CIDs, for which biological assay information is available, are flagged to enable users to rapidly compare cytotoxicity values in different cell lines. By coalescing these elements, SMCVdb provides a data-driven platform that facilitates the streamlined selection and prioritization of chemical compounds while also fostering the exploration of associations between these compounds and toxicity. The Also, a dedicated update tab is present in SMCVdb to keep users informed of new data and functionalities.

### Web interface

**Figure 2.**
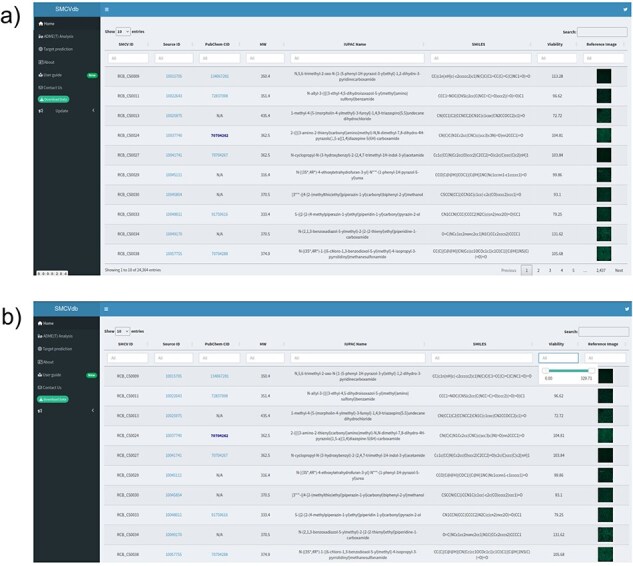
The homepage of the database: (a) SMCVdb homepage displays various options such as ADME(T) Analysis, Target prediction, About, and Contact Us; (b) Displaying the option to use the sliding button to set the range of viability percentage score.

The home page of the database is divided into three sections (Fig. 2a) the header, the sidebar, and the main section. The header contains the title of the interface and a link to the developer’s Twitter account for updates. The sidebar provides users with relevant information and different section for recommended online servers for ADME(T) analysis, such as ADMETlab 2.0 and SwissADME, as well as SwissTargetPrediction tab for target prediction with link to recommended server link. To use these servers, the SMILES notation of all the compounds is also shared. The database also has an example-based tutorial accessible from the User Guide link in the side panel of the database. This tutorial provides case-based guidance, with specific examples, on utilizing the database effectively.

### Search for information

The SMCVdb was developed to provide researchers with a toxicity profile of 24 361 compounds. The database contains essential information about each compound, such as identification (ID), molecular weight, compound name, smile notation, and the hyperlink to the source website. The source page provides comprehensive chemical description and pricing information for researchers to easily find and retrieve specific compounds of interest using unique IDs. Users can access additional chemical information by clicking the link under Source ID option. The database has the option to select compounds based on specific viability range as well as molecular weight range. The users can use the slider at the home page to set a range of molecular weight or viability percentage score for visualization (Fig. 2b).

### The associations between entities

**Figure 3.**
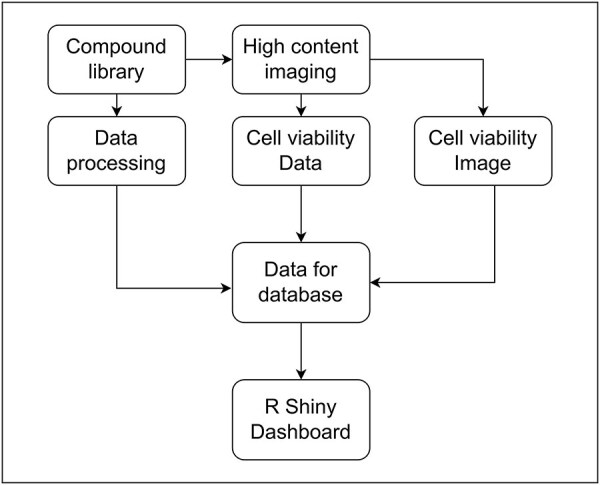
Flowchart showing the process of data collection and curation for the database: Association between the entities and data in SMCVdb.

To investigate the relationship between small molecule compounds and cellular viability, we used HCI to assess the toxicity of compounds against a screen of human cells. The data generated from HCI enabled us to evaluate relationships between compound properties and their effects on cell viability (as shown in Fig. 3). Through SMCVdb, users can examine relationships between compound viability profiles and factors such as molecular weight, compound structure, and specific cell types. This facilitates the identification of compounds with size-dependent toxicological effects and supports the investigation of structure–activity relationships, offering valuable insights for the design of safer and more effective small molecule therapeutics.

### Common compatibility

The SMCVdb is a valuable resource for researchers interested in studying chemical toxicity. By providing toxicity profiles for a wide range of compounds, the database allows researchers to compare and analyze patterns and levels of toxicity across different molecules. This information can help identify common association trends or distinguish compounds with unique toxicity profiles. Thus, the SMCVdb facilitates the evaluation of compound compatibility and helps to identify potentially toxic or safe compounds for various applications. This database also provides the facility of cross-referencing to other publicly available servers for further physiochemical profiling.

### Data analysis and visualization

**Figure 4.**
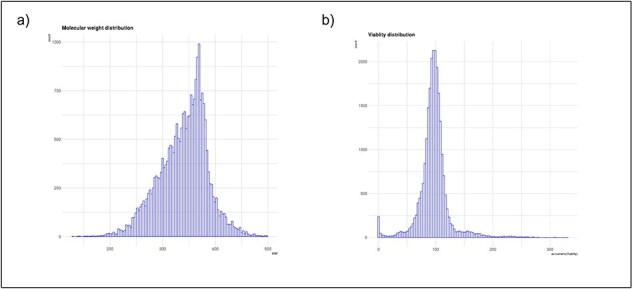
Basic statistics of SMCVdb database: (a) A bar graph showing the distribution of cell viability scores for the small molecules in your database; (b) A bar graph showing the distribution of molecular weight.

To facilitate data analysis and visualization, SMCVdb includes user-friendly tools and interactive visualizations. Researchers can query the database based on specific criteria, such as compound name, molecular weight range, and viability percentage. Figure 4a and b shows the distribution of cell viability scores and molecular weight. The database also provides options for filtering and visualizing data, allowing easy exploration of compound viability profiles and comparisons between different compounds. Visualization tools allow researchers to identify trends, patterns, and potential relationships within a dataset to support further exploration and hypothesis generation.

## Conclusion

The SMCVdb is a comprehensive resource that combines toxicity profiling data with chemical descriptions and pricing information. This integration provides researchers with a resource to obtain information regarding the effects of small molecule compounds on cellular viability. SMCVdb provides an extensive collection of compound viability profiles, including vital information such as viability percentage, molecular weight, compound name, source link, etc. Its user-friendly web interface allows easy searching, filtering, and graphical display of data, allowing researchers to query the database based on specific criteria. It is an invaluable resource for chemical safety assessment and environmental toxicology researchers. It enhances the ability of researchers to make informed decisions on compound selection, lead optimization, and cost-effective purchases. The SMCVdb stands ready to expedite research and facilitate informed decision-making.

In conclusion, the SMCVdb is a valuable tool for researchers who study the effects of small molecule compounds on cellular viability. The database provides a comprehensive and user-friendly platform for accessing toxicity profiling data, chemical descriptions, and pricing information. SMCVdb stands ready to accelerate research, facilitate informed decision-making, and contribute cost-effective practices in drug discovery and compound evaluation.

## Future prospects

The SMCVdb has tremendous potential for further development and improvement. The database can be expanded to include a wider range of small molecules and cell types, and be combined with other multi-omics data and *in vivo* toxicity data. The SMCVdb provides scope to develop predictive algorithms using machine learning for toxic compounds. The database will also encourage other scientific teams to contribute cellular viability information for other cells types and compound libraries. The SMCVdb can also be enhanced by integrating databases and related features in addition to improving the database by developing interactive diagrams and data analysis tools. The proposed enhancements will make the SMCVdb more comprehensive, informative, and user-friendly for researchers in the fields of drug discovery, toxicology, and drug safety.

## Supplementary Material

baae100_Supp

## Data Availability

The datasets generated and analyzed in this study are openly accessible at:http://smcvdb.rcb.ac.in:4321/. The interface is free for all users, with no requirements of login or licence.
