# Supplementary material for: SMCVdb: a database of experimental cellular toxicity information for drug candidate molecules
Source: Database (Oxford). 2024 Oct 18;2024:baae100. doi: 10.1093/database/baae100 (PMC11488516; doi:10.1093/database/baae100)

SMCVdbTutorial

Case 1: Searching the database

Case 2: Filtering compounds based on molecular weight

Case 3: Filtering compounds based on viability value

Case 4 : Downloading microscopy image data of a specific compound

Case 5: Commercial procurement information for a specific compound

Case 6: Downloading compound in molecular docking-supported format

Case 7: Accessing PubChem Information for a specific compound

Case 8: Performing ADME(T) analysis of a selected compound

Case 9: Target prediction for a specific compound

Case 10: User support

Case 1 : Searching the database

Example 1: Using Chemical group name

- For example **Benzofuran**, a key building block in nature, finds itself not only in biologically active natural products but also in synthetic materials. This versatile scaffold boasts a range of potential health benefits, including anti-inflammatory, antimicrobial, antifungal, antidiabetic, pain-relieving, antiparasitic, and antitumor properties.

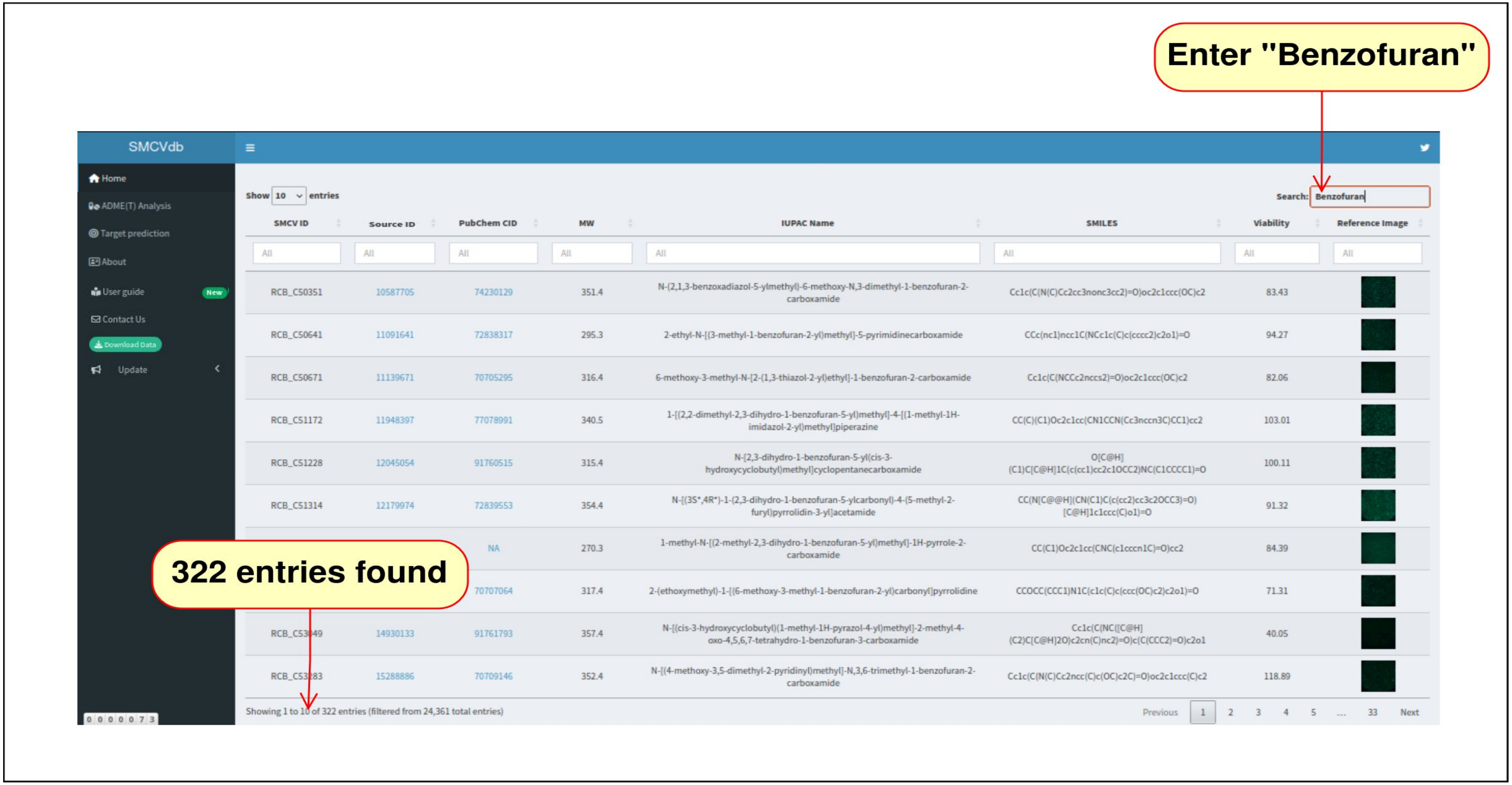

Tutorial main

Case 1 : Searching the database

Example 1: Using Chemical group name

- For example **Pyrazole,** Pyrazole-containing molecules display a broad range of biological activities, including anti-inflammatory, anticonvulsant, anticancer, antiviral, antidepressant, analgesic, antibacterial, antifungal, and selective enzyme inhibition.

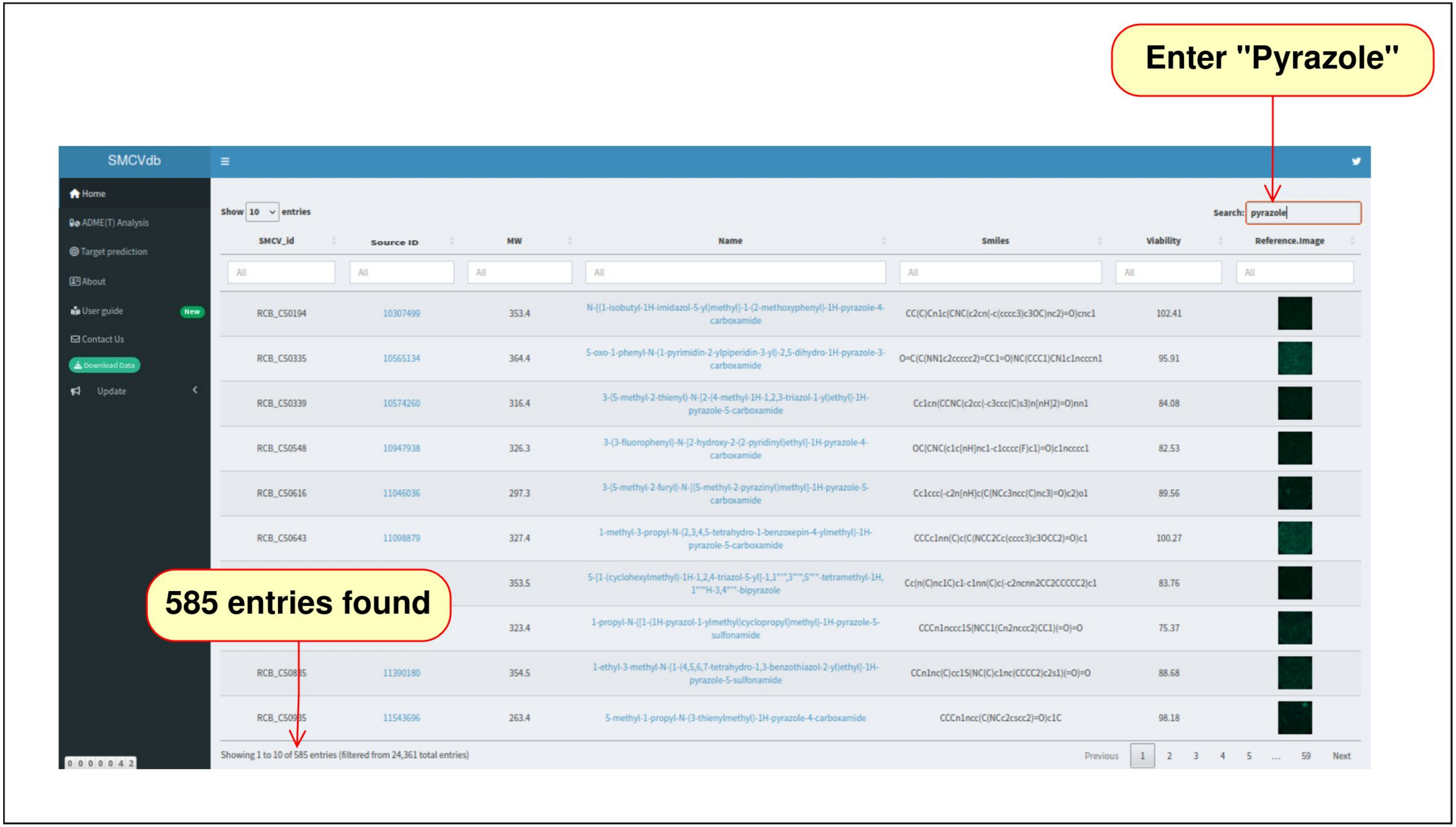

Tutorial main

Case 1 : Searching the database

Example 2: Using other strings related to SMCVdb entries

- Users can search the database using string search, Such as using SMILES. For example "CSCCN(CC1)CCN1C(c1cc(-c2c(CO)cccc2)ccc1)=O"

  When you enter this string, The database will return the matched result.

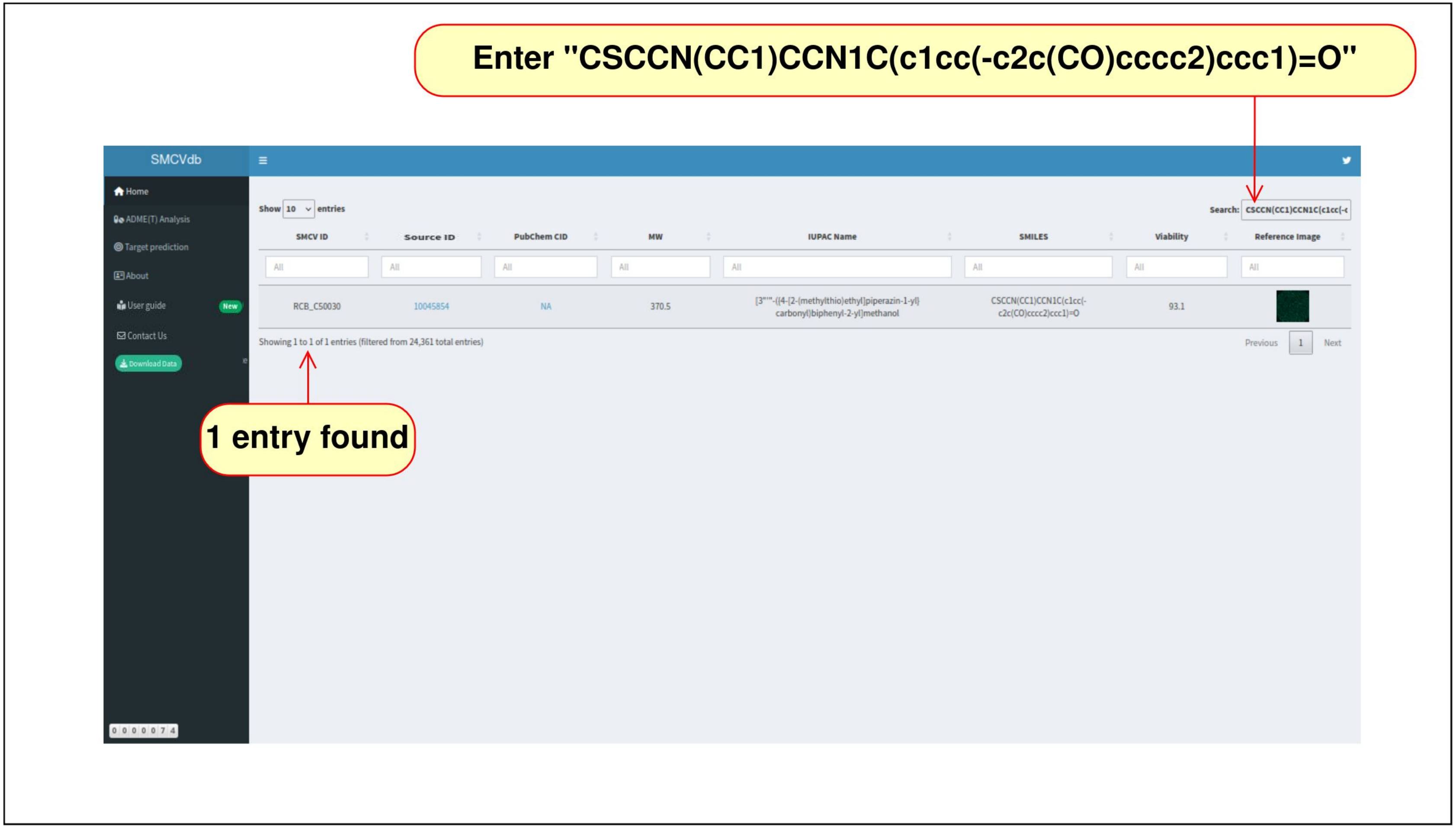

Tutorial main

Case 1 : Searching the database

Example 2: Using other strings related to SMCVdb entries

- Users can search the database using ‘partial’ string search, Such as using SMILES. For example “CSCCN(CC1)”

  When you enter this string, The database will return the matched result.

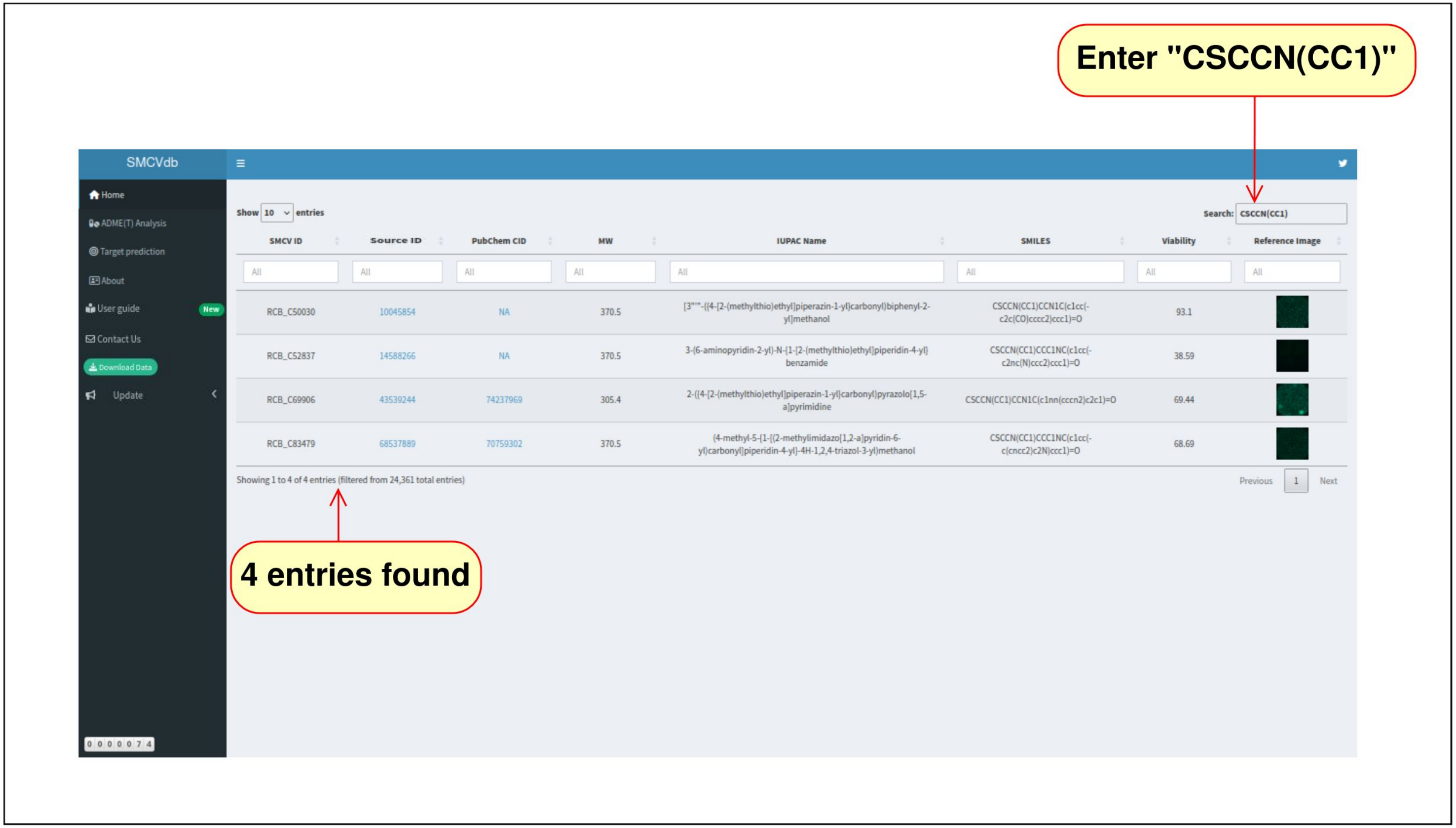

Case 2: Filtering compounds based on molecular weight

Step 1 : Open the home page

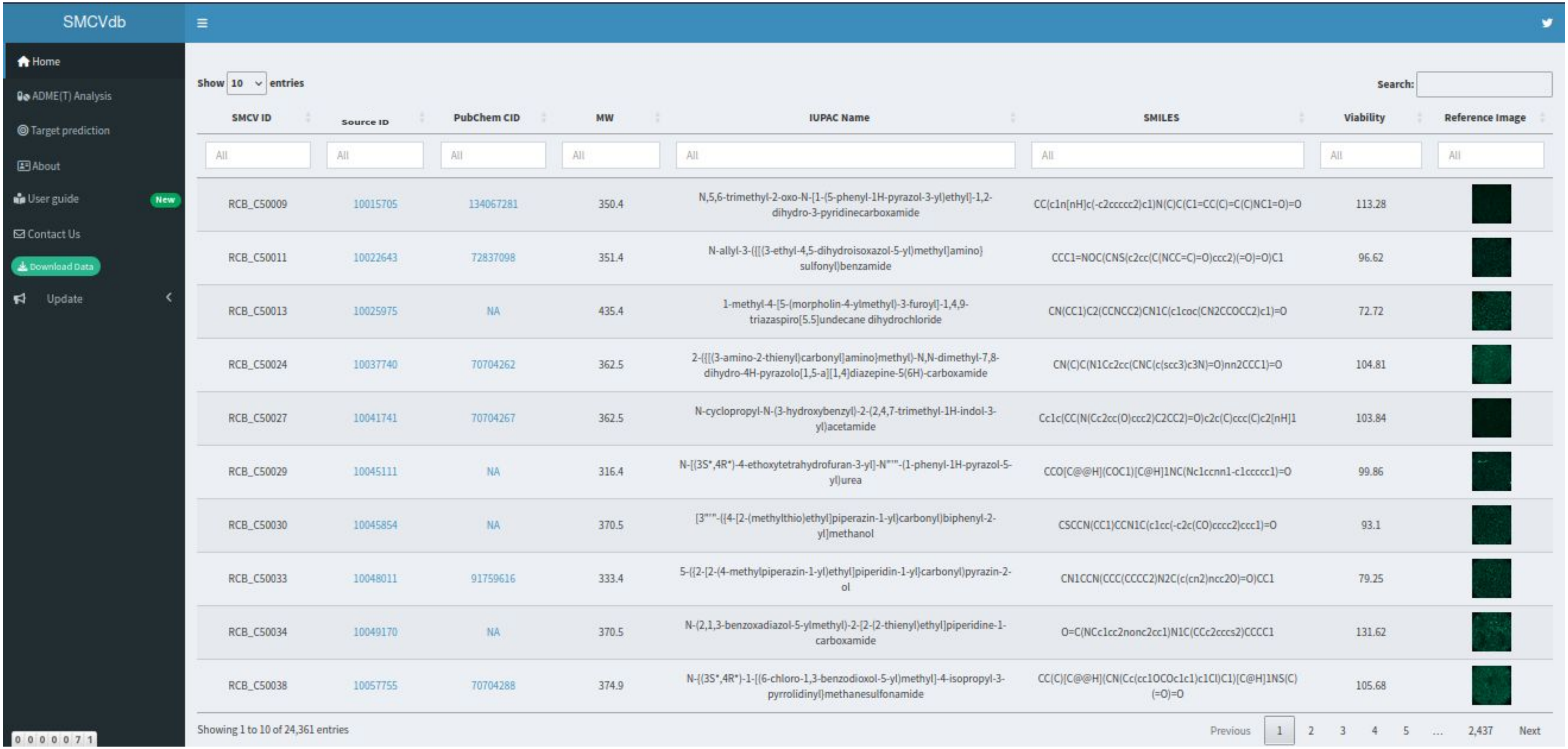

SMCV ID	Source ID	PubChem CID	MW	IUPAC Name	SMILES	Viability	Reference Image
All	All	All	All	All	All	All	All
RCB_C50009	10015705	134067281	350.4	N,5,6-trimethyl-2-oxo-N-[1-(5-phenyl-1H-pyrazol-3-yl)ethyl]-1,2-dihydro-3-pyridinecarboxamide	CC(c1n[nH]c(-c2ccccc2)c1)N(C)C(C1=CC(C)=C(C)NC1=O)=O	113.28	
RCB_C50011	10022643	72837098	351.4	N-allyl-3-({[(3-ethyl-4,5-dihydroisoxazol-5-yl)methyl]amino}sulfonyl)benzamide	CCC1=NOC(CNS(c2cc(C(NCC=C)=O)ccc2)(=O)=O)C1	96.62	
RCB_C50013	10025975	NA	435.4	1-methyl-4-[5-(morpholin-4-ylmethyl)-3-furoyl]-1,4,9-triazaspiro[5.5]undecane dihydrochloride	CN(CC1)C2(CCNCC2)CN1C(c1coc(CN2CCOCC2)c1)=O	72.72	
RCB_C50024	10037740	70704262	362.5	2-({[(3-amino-2-thienyl)carbonyl]amino}methyl)-N,N-dimethyl-7,8-dihydro-4H-pyrazolo[1,5-a][1,4]diazepine-5(6H)-carboxamide	CN(C)C(N1Cc2cc(CNC(c(scc3)c3N)=O)nn2CCC1)=O	104.81	
RCB_C50027	10041741	70704267	362.5	N-cyclopropyl-N-(3-hydroxybenzyl)-2-(2,4,7-trimethyl-1H-indol-3-yl)acetamide	Cc1c(CC(N(Cc2cc(O)ccc2)C2CC2)=O)c2c(C)ccc(C)c2[nH]1	103.84	
RCB_C50029	10045111	NA	316.4	N-[(3S*,4R*)-4-ethoxytetrahydrofuran-3-yl]-N"'"-(1-phenyl-1H-pyrazol-5-yl)urea	CCO[C@@H](COC1)[C@H]1NC(Nc1ccnn1-c1ccccc1)=O	99.86	
RCB_C50030	10045854	NA	370.5	[3"'"-({4-[2-(methylthio)ethyl]piperazin-1-yl}carbonyl)biphenyl-2-yl]methanol	CSCCN(CC1)CCN1C(c1cc(-c2c(CO)cccc2)ccc1)=O	93.1	
RCB_C50033	10048011	91759616	333.4	5-({2-[2-(4-methylpiperazin-1-yl)ethyl]piperidin-1-yl}carbonyl)pyrazin-2-ol	CN1CCN(CCC(CCCC2)N2C(c(cn2)ncc2O)=O)CC1	79.25	
RCB_C50034	10049170	NA	370.5	N-(2,1,3-benzoxadiazol-5-ylmethyl)-2-[2-(2-thienyl)ethyl]piperidine-1-carboxamide	O=C(NCc1cc2nonc2cc1)N1C(CCc2cccs2)CCCC1	131.62	
RCB_C50038	10057755	70704288	374.9	N-{(3S*,4R*)-1-[(6-chloro-1,3-benzodioxol-5-yl)methyl]-4-isopropyl-3-pyrrolidinyl}methanesulfonamide	CC(C)[C@@H](CN(Cc(cc1OCOc1c1)c1Cl)C1)[C@H]1NS(C)(=O)=O	105.68	

Case 2: Filtering compounds based on molecular weight

Step 2 : Click on the MW slider

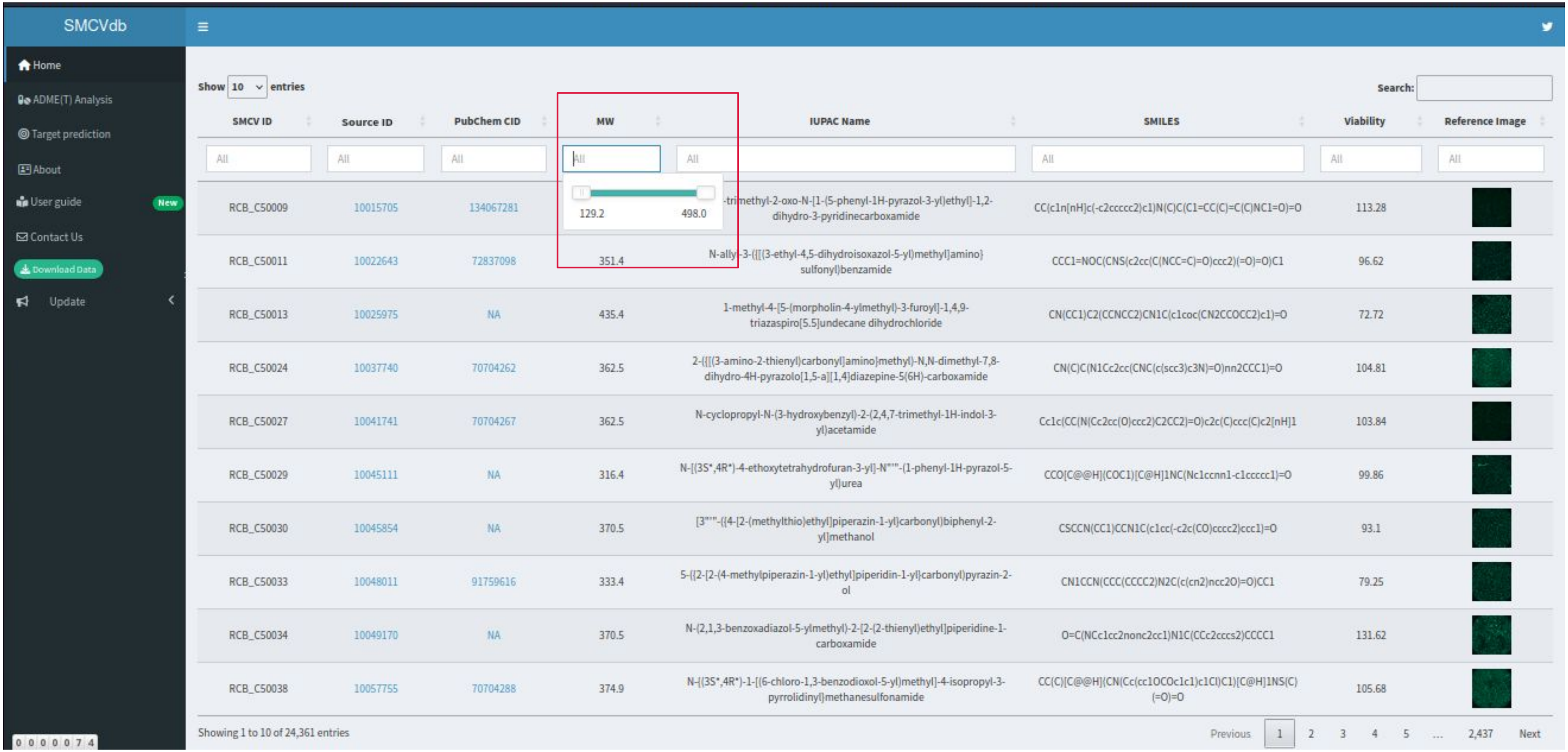

Case 2: Filtering compounds based on molecular weight

Step 3 : Select the range

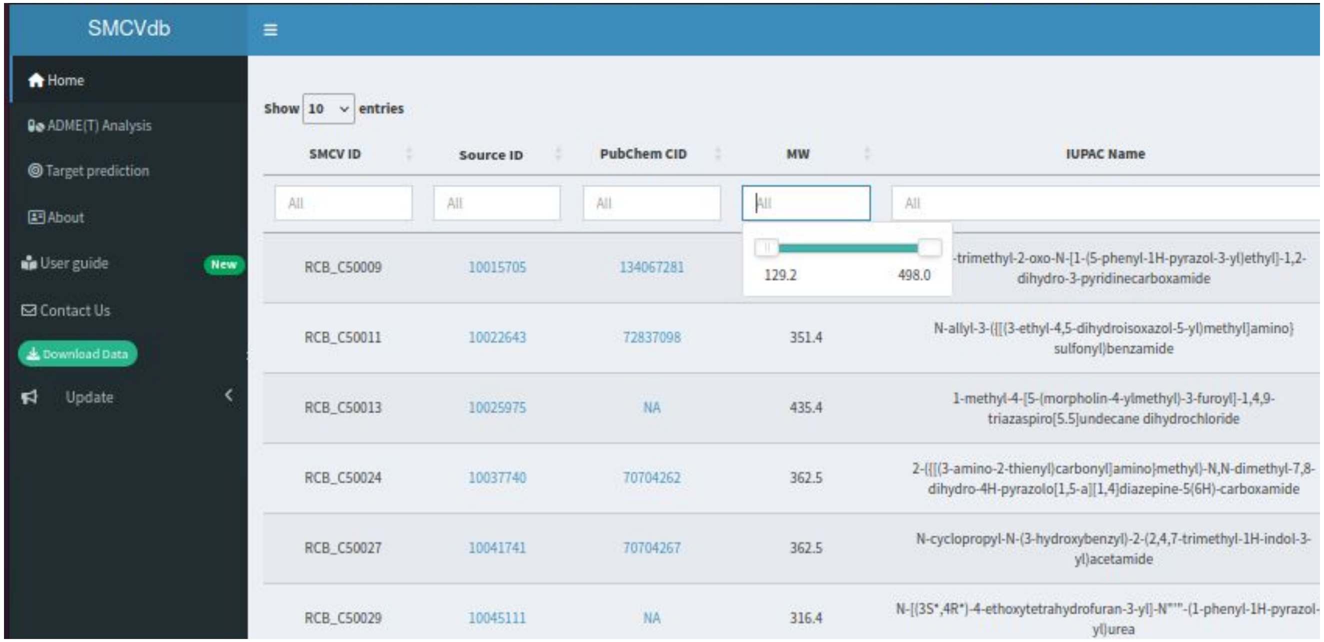

Note : For manual entry in MW use “100**...**150” format

Case 2: Filtering compounds based on molecular weight

Step 4 : Result page will appear

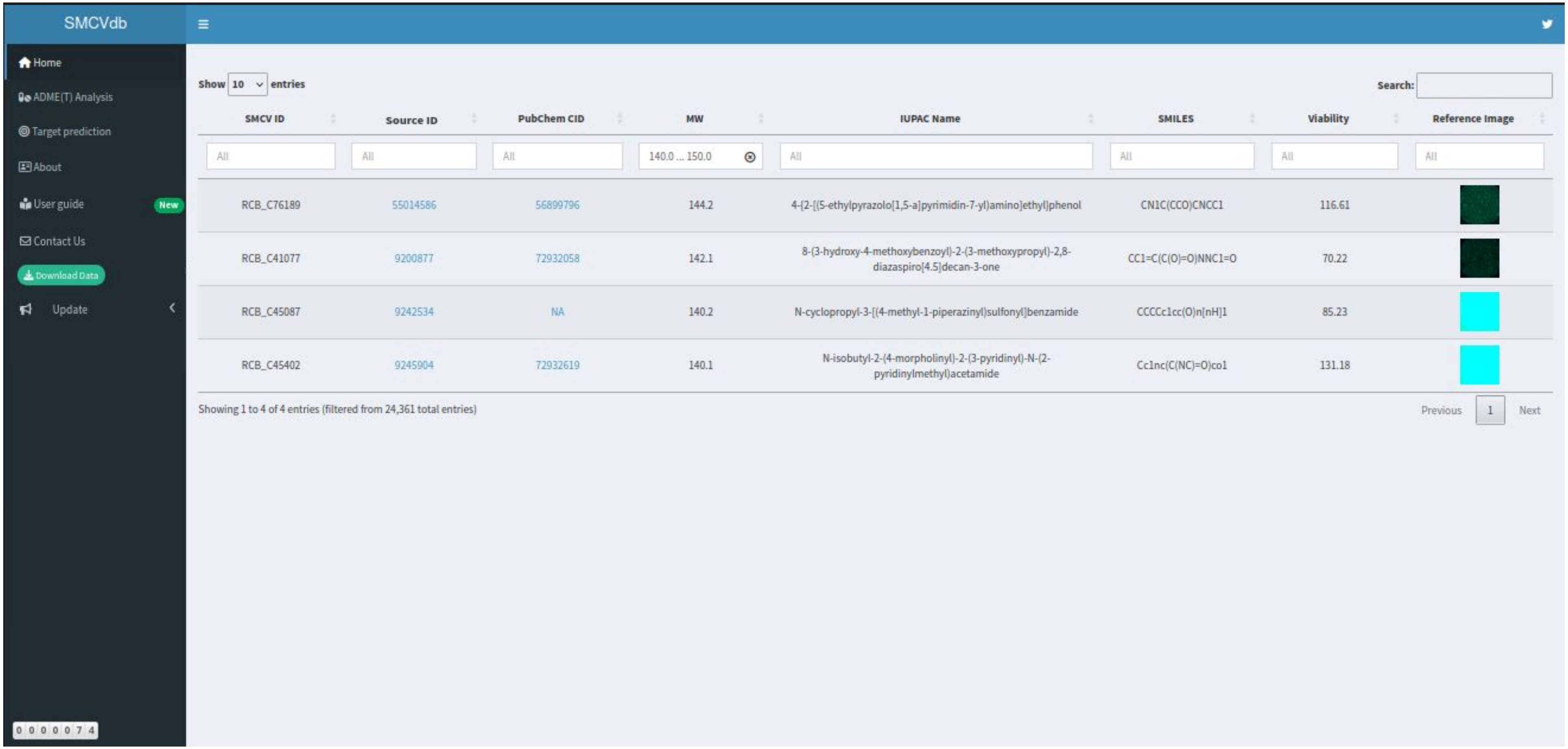

SMCV ID	Source ID	PubChem CID	MW	IUPAC Name	SMILES	Viability	Reference Image
All	All	All	140.0 ... 150.0	All	All	All	All
RCB_C76189	55014586	56899796	144.2	4-[2-[(5-ethylpyrazolo[1,5-a]pyrimidin-7-yl)amino]ethyl]phenol	CN1C(CCO)CNCC1	116.61	
RCB_C41077	9200877	72932058	142.1	8-(3-hydroxy-4-methoxybenzoyl)-2-(3-methoxypropyl)-2,8-diazaspiro[4.5]decan-3-one	CC1=C(C(O)=O)NNC1=O	70.22	
RCB_C45087	9242534	NA	140.2	N-cyclopropyl-3-[(4-methyl-1-piperazinyl)sulfonyl]benzamide	CCCCc1cc(O)n[nH]1	85.23	
RCB_C45402	9245904	72932619	140.1	N-isobutyl-2-(4-morpholinyl)-2-(3-pyridinyl)-N-(2-pyridinylmethyl)acetamide	Cc1nc(C(NC)=O)co1	131.18	

Case 3: Filtering compounds within a range of viability information

Step 1 : Open the home page

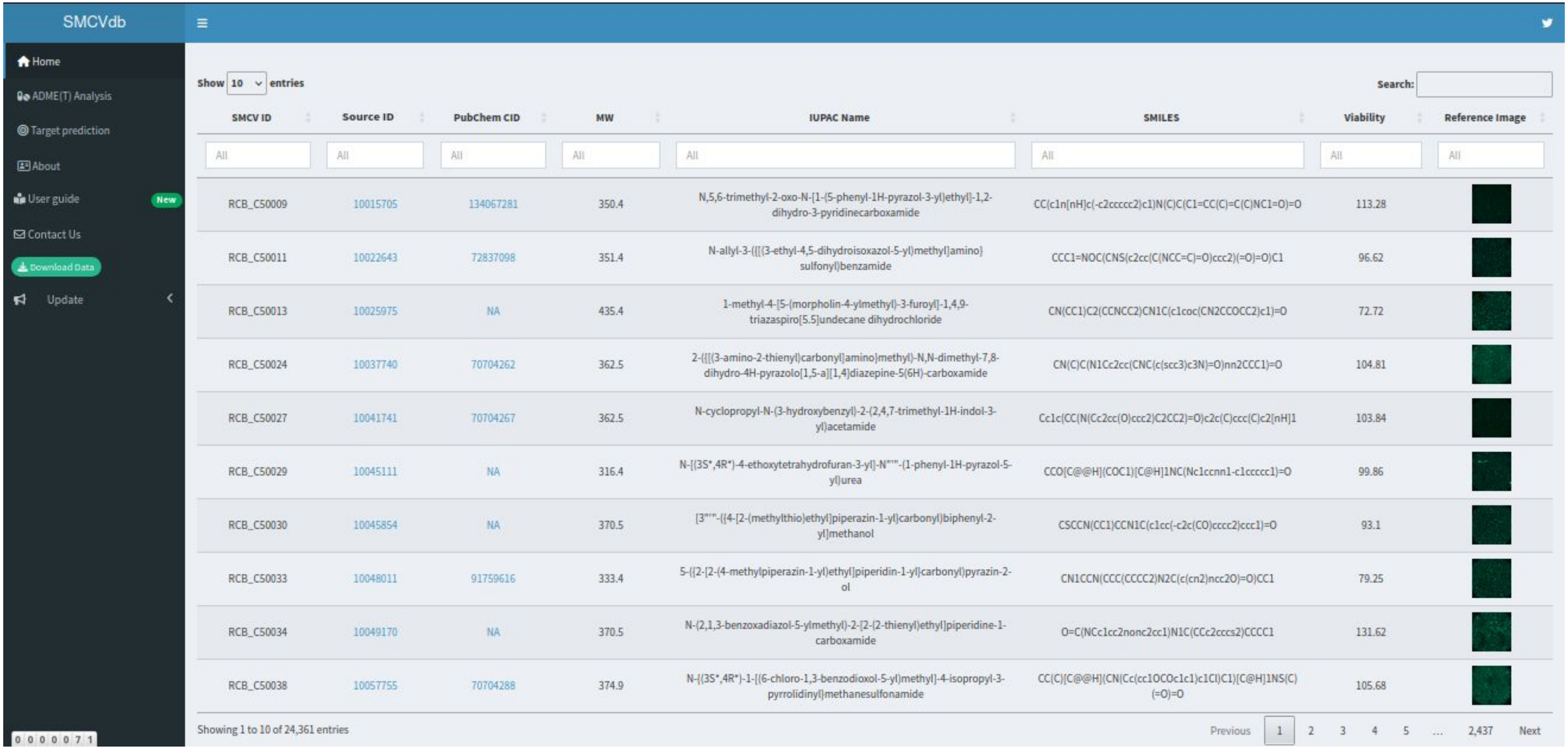

Case 3: Filtering compounds within a range of viability information

Step 2 : Click on the Viability slider

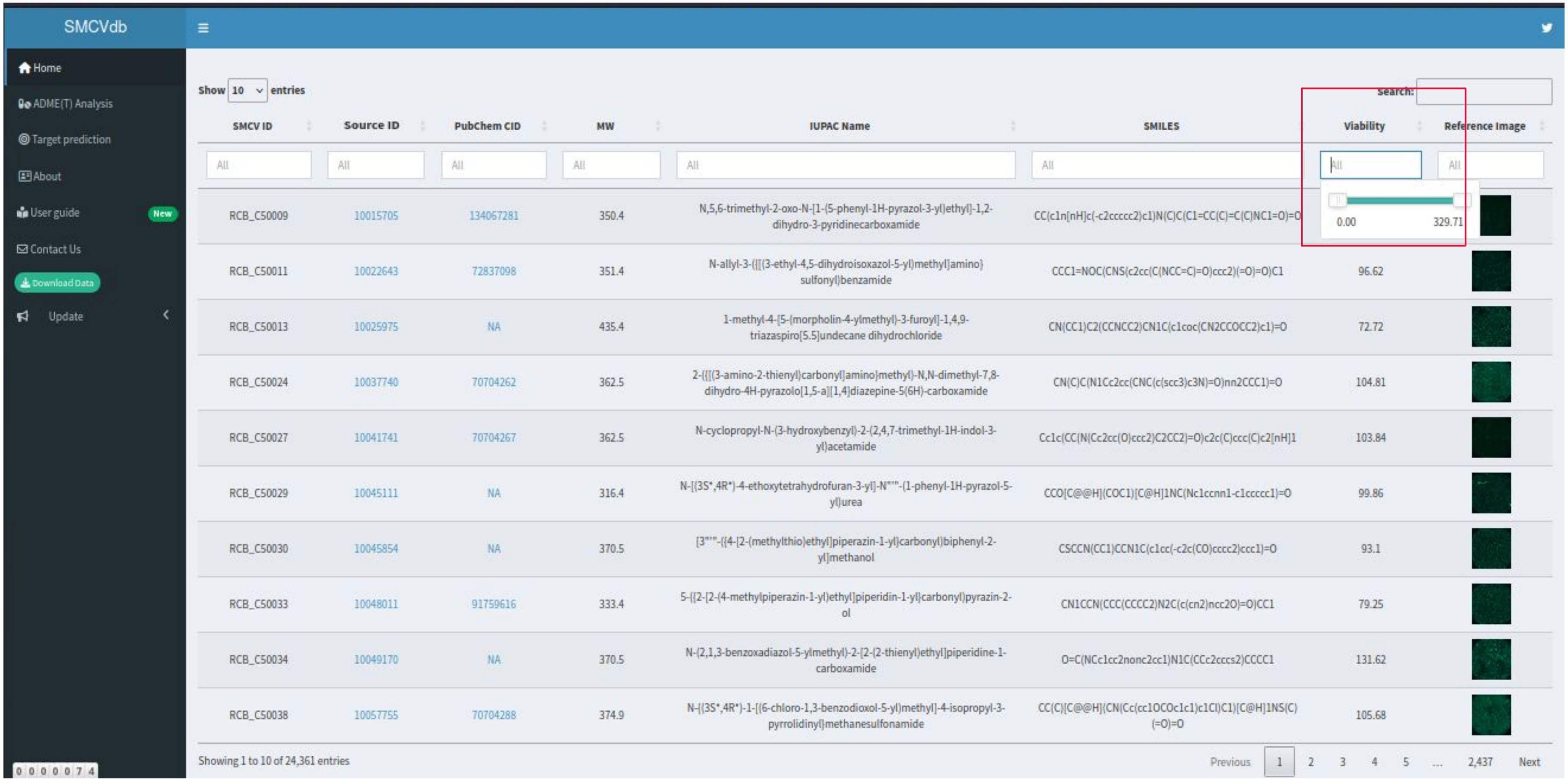

Case 3: Filtering compounds within a range of viability information

Step 3 : Select the range

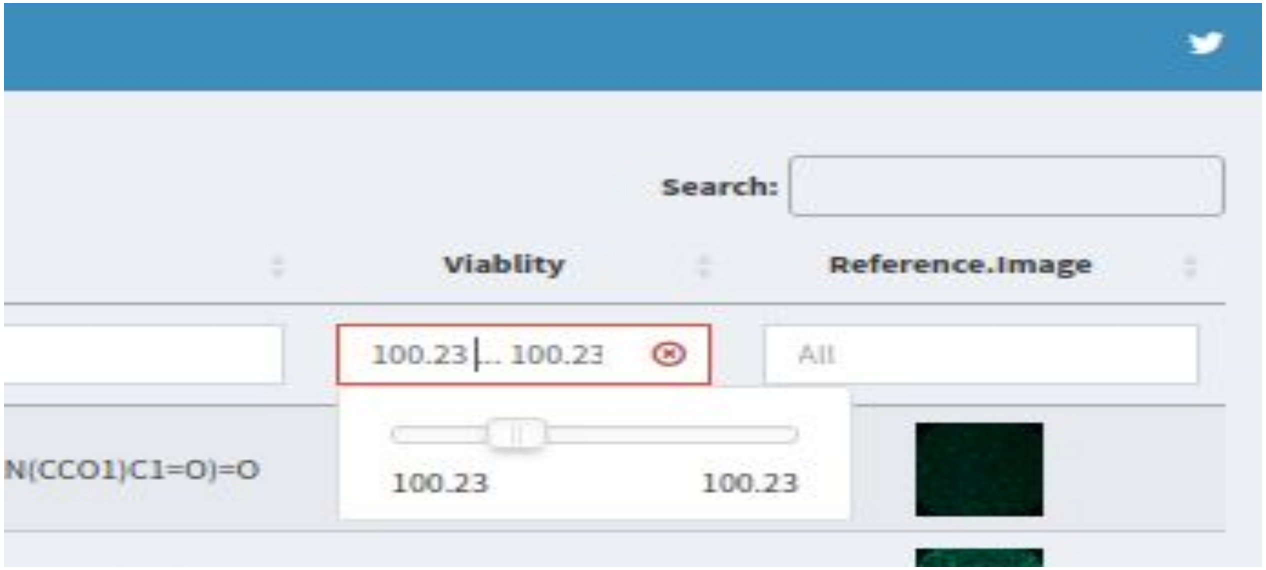

Note : For manual entry of range use “100**...**150” format

Case 3: Filtering compounds within a range of viability information

Step 4 : Result will appear

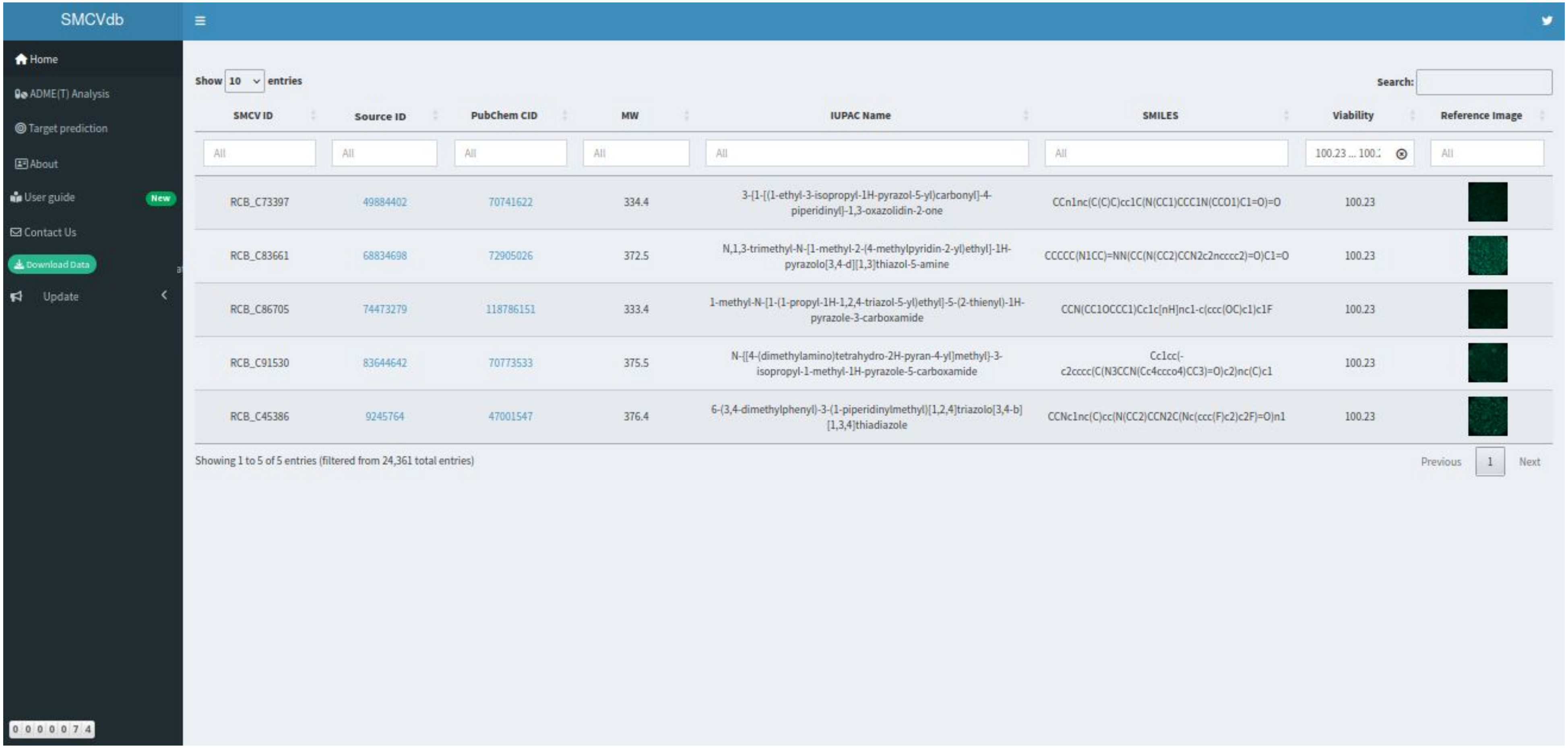

Case 4 : Downloading microscopy image data of a specific compound

Step 1 : Choose your compound using case 1-3

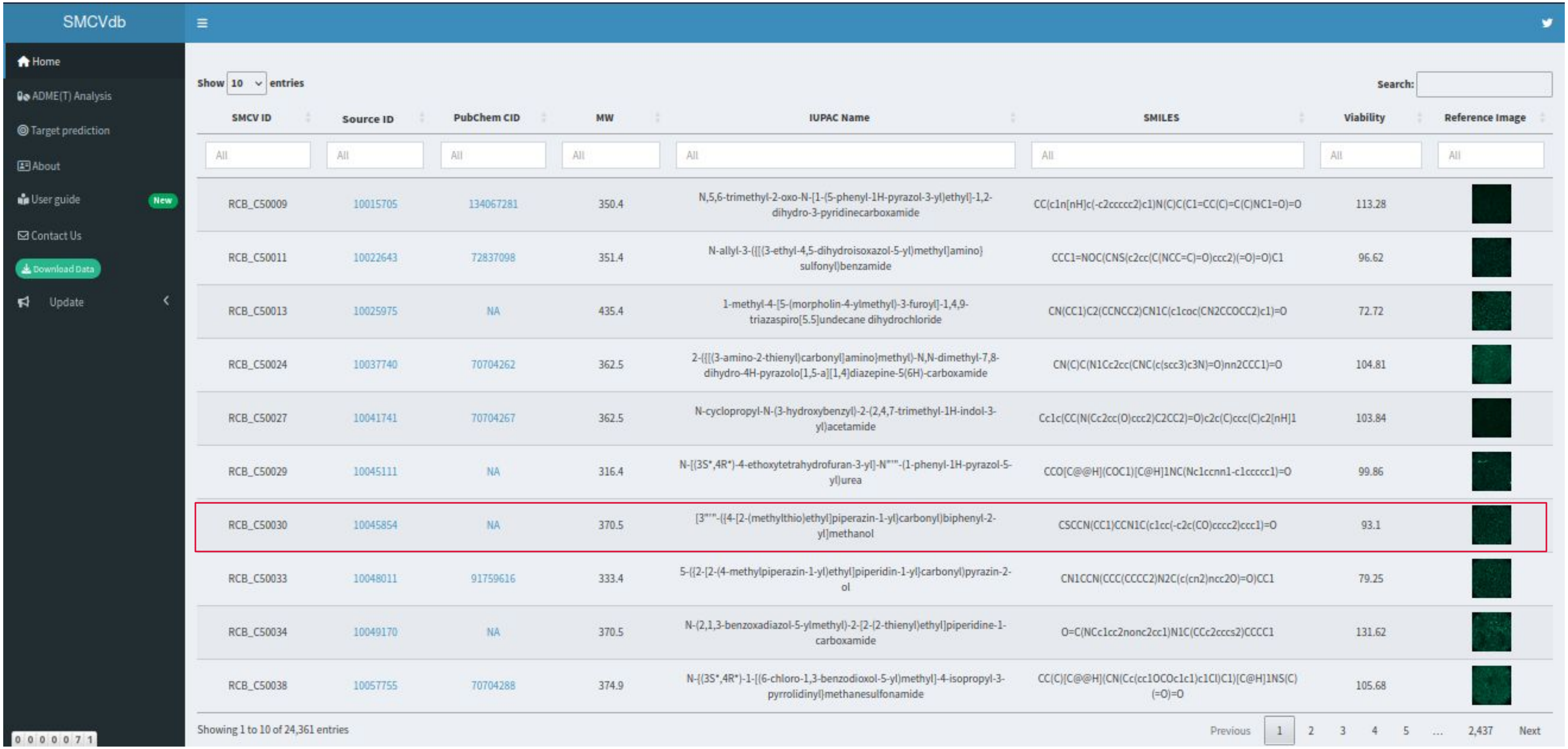

SMCV ID	Source ID	PubChem CID	MW	IUPAC Name	SMILES	Viability	Reference Image
All	All	All	All	All	All	All	All
RCB_C50009	10015705	134067281	350.4	N,5,6-trimethyl-2-oxo-N-[1-(5-phenyl-1H-pyrazol-3-yl)ethyl]-1,2-dihydro-3-pyridinecarboxamide	CC(c1n[nH]c(-c2ccccc2)c1)N(C)C(C1=CC(C)=C(C)NC1=O)=O	113.28	
RCB_C50011	10022643	72837098	351.4	N-allyl-3-({[(3-ethyl-4,5-dihydroisoxazol-5-yl)methyl]amino}sulfonyl)benzamide	CCC1=NOC(CNS(c2cc(C(NCC=C)=O)ccc2)(=O)=O)C1	96.62	
RCB_C50013	10025975	NA	435.4	1-methyl-4-[5-(morpholin-4-ylmethyl)-3-furoyl]-1,4,9-triazaspiro[5.5]undecane dihydrochloride	CN(CC1)C2(CCNCC2)CN1C(c1coc(CN2CCOCC2)c1)=O	72.72	
RCB_C50024	10037740	70704262	362.5	2-({[(3-amino-2-thienyl)carbonyl]amino}methyl)-N,N-dimethyl-7,8-dihydro-4H-pyrazolo[1,5-a][1,4]diazepine-5(6H)-carboxamide	CN(C)C(N1Cc2cc(CNC(c(scc3)c3N)=O)nn2CCC1)=O	104.81	
RCB_C50027	10041741	70704267	362.5	N-cyclopropyl-N-(3-hydroxybenzyl)-2-(2,4,7-trimethyl-1H-indol-3-yl)acetamide	Cc1c(CC(N(Cc2cc(O)ccc2)C2CC2)=O)c2c(C)ccc(C)c2[nH]1	103.84	
RCB_C50029	10045111	NA	316.4	N-[(3S*,4R*)-4-ethoxytetrahydrofuran-3-yl]-N"'"-(1-phenyl-1H-pyrazol-5-yl)urea	CCO[C@@H](COC1)[C@H]1NC(Nc1ccnn1-c1ccccc1)=O	99.86	
RCB_C50030	10045854	NA	370.5	[3"'"-({4-[2-(methylthio)ethyl]piperazin-1-yl}carbonyl)biphenyl-2-yl]methanol	CSCCN(CC1)CCN1C(c1cc(-c2c(CO)cccc2)ccc1)=O	93.1	
RCB_C50033	10048011	91759616	333.4	5-({2-[2-(4-methylpiperazin-1-yl)ethyl]piperidin-1-yl}carbonyl)pyrazin-2-ol	CN1CCN(CCC(CCCC2)N2C(c(cn2)ncc2O)=O)CC1	79.25	
RCB_C50034	10049170	NA	370.5	N-(2,1,3-benzoxadiazol-5-ylmethyl)-2-[2-(2-thienyl)ethyl]piperidine-1-carboxamide	O=C(NCc1cc2nonc2cc1)N1C(CCc2cccs2)CCCC1	131.62	
RCB_C50038	10057755	70704288	374.9	N-{(3S*,4R*)-1-[(6-chloro-1,3-benzodioxol-5-yl)methyl]-4-isopropyl-3-pyrrolidinyl}methanesulfonamide	CC(C)[C@@H](CN(Cc(cc1OCOc1c1)c1Cl)C1)[C@H]1NS(C)(=O)=O	105.68	

Tutorial main

Case 4 : Downloading microscopy image data of a specific compound

Step 2 : Click on the reference image link

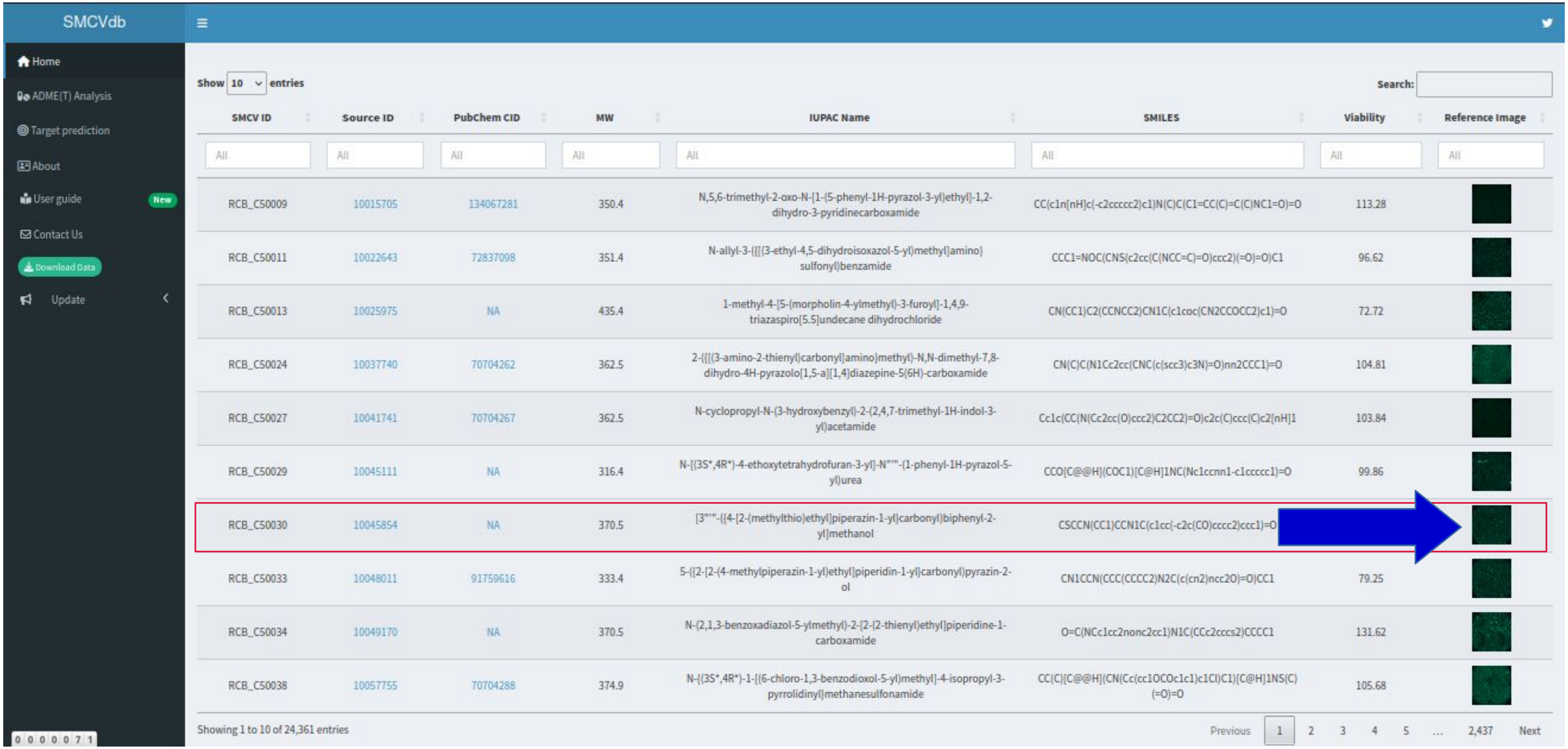

SMCV ID	Source ID	PubChem CID	MW	IUPAC Name	SMILES	Viability	Reference Image
RCB_C50009	10015705	134067281	350.4	N,5,6-trimethyl-2-oxo-N-[1-(5-phenyl-1H-pyrazol-3-yl)ethyl]-1,2-dihydro-3-pyridinecarboxamide	CC(c1n[nH]c(-c2ccccc2)c1)N(C)C(C1=CC(C)=C(C)NC1=O)=O	113.28	
RCB_C50011	10022643	72837098	351.4	N-allyl-3-({[(3-ethyl-4,5-dihydroisoxazol-5-yl)methyl]amino}sulfonyl)benzamide	CCC1=NOC(CNS(c2cc(C(NCC=C)=O)ccc2)(=O)=O)C1	96.62	
RCB_C50013	10025975	NA	435.4	1-methyl-4-[5-(morpholin-4-ylmethyl)-3-furoyl]-1,4,9-triazaspiro[5.5]undecane dihydrochloride	CN(CC1)C2(CCNCC2)CN1C(c1coc(CN2CCOCC2)c1)=O	72.72	
RCB_C50024	10037740	70704262	362.5	2-({[(3-amino-2-thienyl)carbonyl]amino}methyl)-N,N-dimethyl-7,8-dihydro-4H-pyrazolo[1,5-a][1,4]diazepine-5(6H)-carboxamide	CN(C)C(N1Cc2cc(CNC(c(scc3)c3N)=O)nn2CCC1)=O	104.81	
RCB_C50027	10041741	70704267	362.5	N-cyclopropyl-N-(3-hydroxybenzyl)-2-(2,4,7-trimethyl-1H-indol-3-yl)acetamide	Cc1c(CC(N(Cc2cc(O)ccc2)C2CC2)=O)c2c(C)ccc(C)c2[nH]1	103.84	
RCB_C50029	10045111	NA	316.4	N-[(3S*,4R*)-4-ethoxytetrahydrofuran-3-yl]-N"'"-(1-phenyl-1H-pyrazol-5-yl)urea	CCO[C@@H](COC1)[C@H]1NC(Nc1ccnn1-c1ccccc1)=O	99.86	
RCB_C50030	10045854	NA	370.5	[3"'"-({4-[2-(methylthio)ethyl]piperazin-1-yl}carbonyl)biphenyl-2-yl]methanol	CSCCN(CC1)CCN1C(c1cc(-c2c(CO)cccc2)ccc1)=O	[illegible]	
RCB_C50033	10048011	91759616	333.4	5-({2-[2-(4-methylpiperazin-1-yl)ethyl]piperidin-1-yl}carbonyl)pyrazin-2-ol	CN1CCN(CCC(CCCC2)N2C(c(cn2)ncc2O)=O)CC1	79.25	
RCB_C50034	10049170	NA	370.5	N-(2,1,3-benzoxadiazol-5-ylmethyl)-2-[2-(2-thienyl)ethyl]piperidine-1-carboxamide	O=C(NCc1cc2nonc2cc1)N1C(CCc2cccs2)CCCC1	131.62	
RCB_C50038	10057755	70704288	374.9	N-{(3S*,4R*)-1-[(6-chloro-1,3-benzodioxol-5-yl)methyl]-4-isopropyl-3-pyrrolidinyl}methanesulfonamide	CC(C)[C@@H](CN(Cc(cc1OCOc1c1)c1Cl)C1)[C@H]1NS(C)(=O)=O	105.68	

Case 4 : Downloading microscopy image data of a specific compound

Step 3 : A pop up window will appear

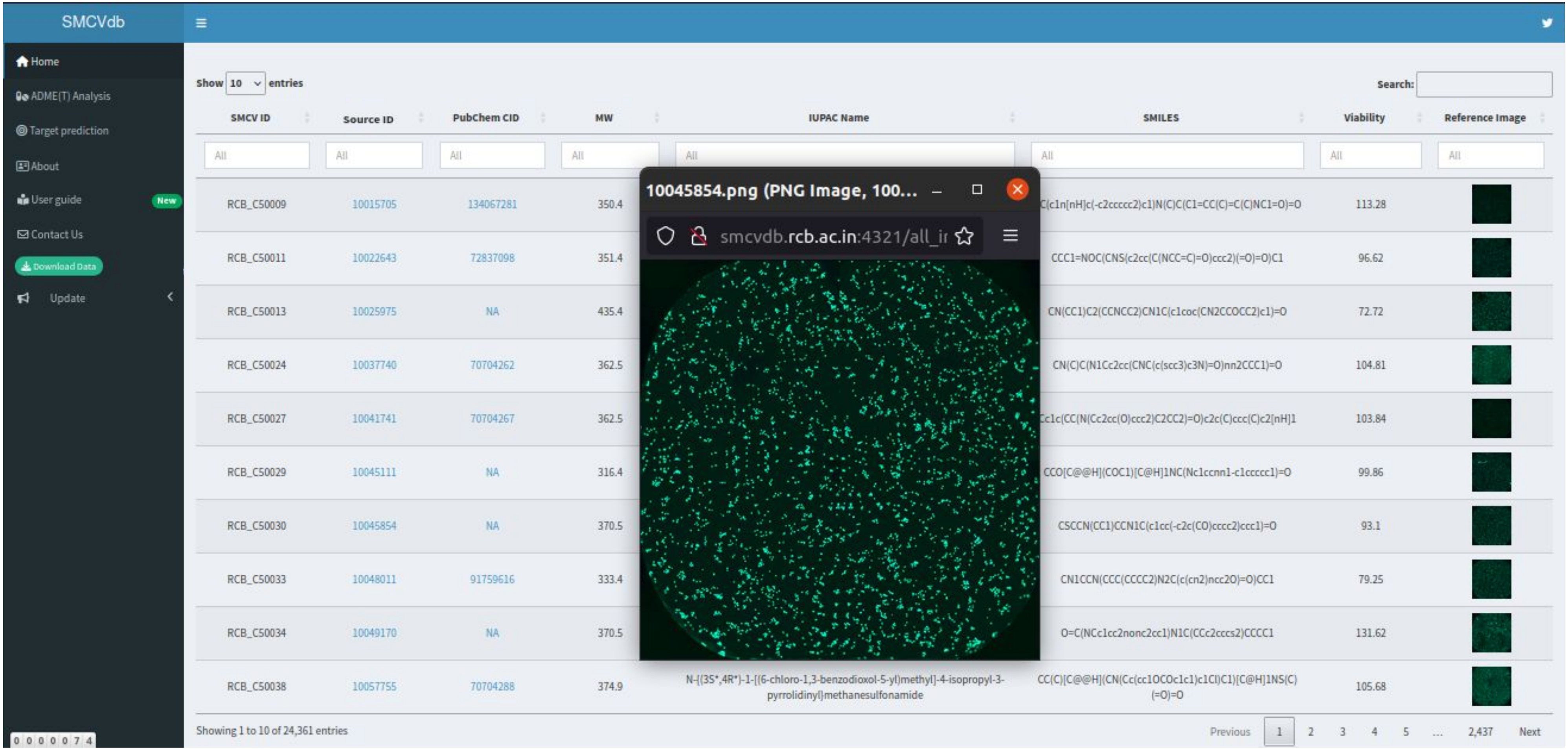

Case 4 : Downloading microscopy image data of a specific compound

Step 4 : Save the image using right click

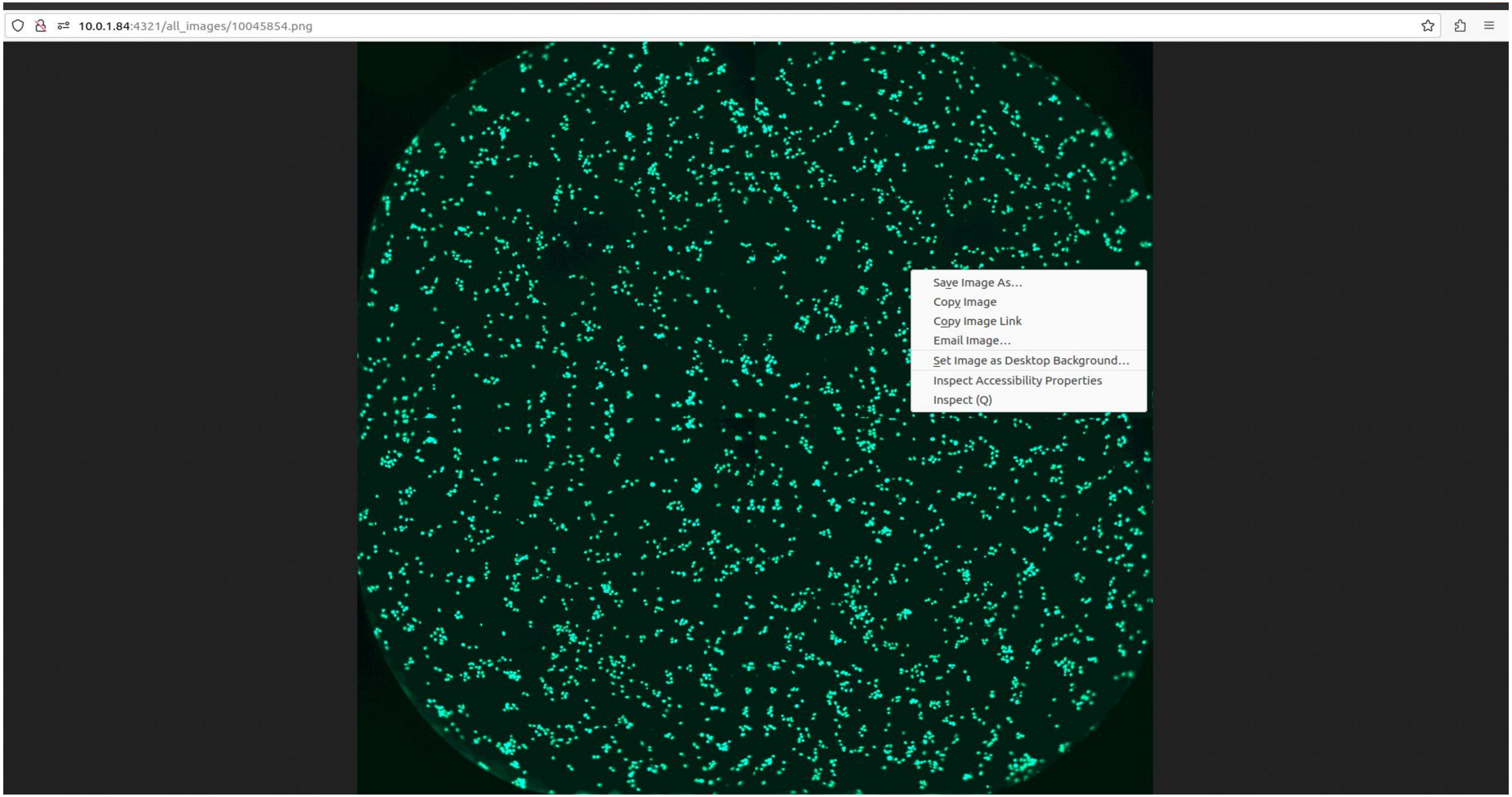

Case 5: Commercial procurement information for a specific compound

Step 1 : Choose your compound using case 1-3

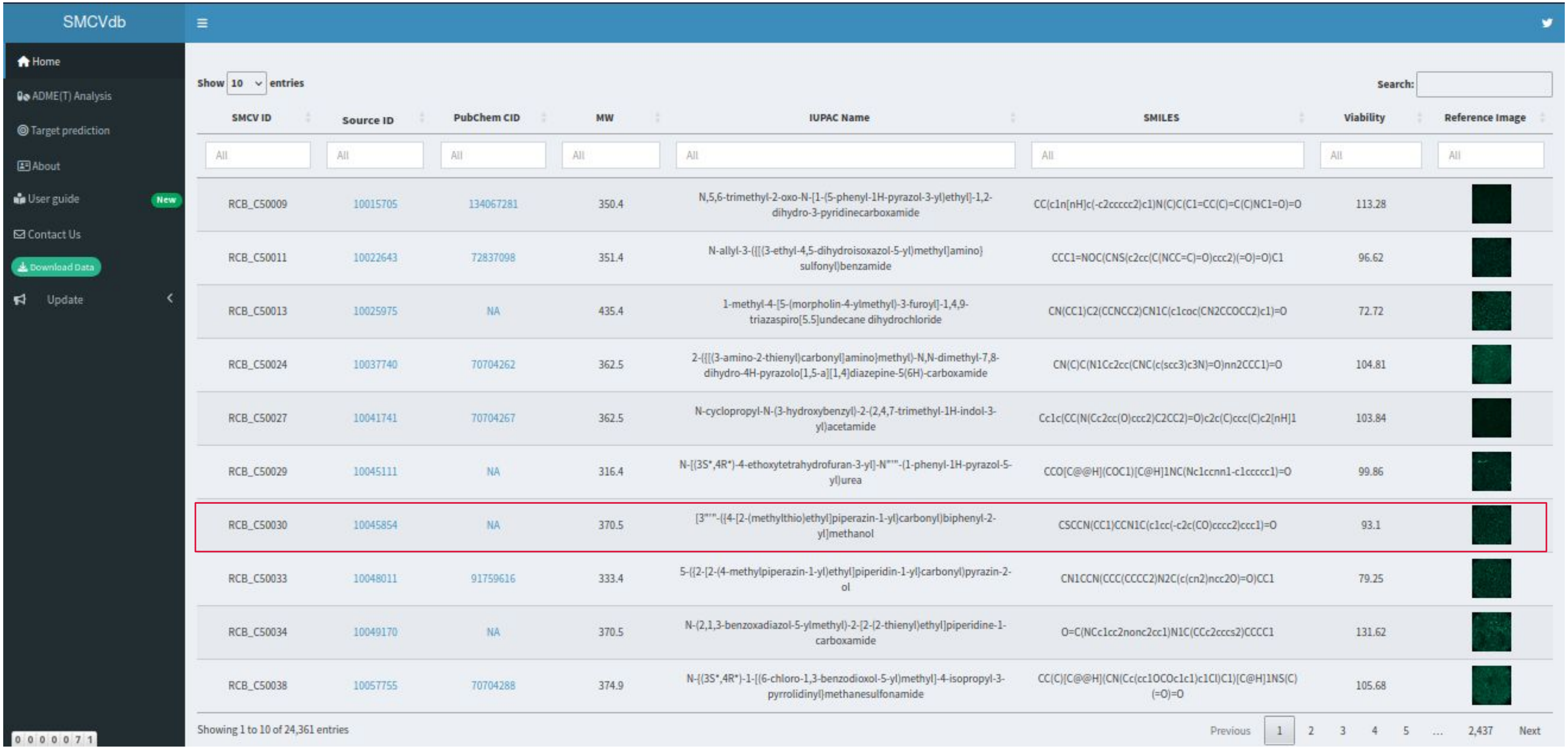

SMCV ID	Source ID	PubChem CID	MW	IUPAC Name	SMILES	Viability	Reference Image
RCB_C50009	10015705	134067281	350.4	N,5,6-trimethyl-2-oxo-N-[1-(5-phenyl-1H-pyrazol-3-yl)ethyl]-1,2-dihydro-3-pyridinecarboxamide	CC(c1n[nH]c(-c2ccccc2)c1)N(C)C(C1=CC(C)=C(C)NC1=O)=O	113.28	
RCB_C50011	10022643	72837098	351.4	N-allyl-3-({[(3-ethyl-4,5-dihydroisoxazol-5-yl)methyl]amino}sulfonyl)benzamide	CCC1=NOC(CNS(c2cc(C(NCC=C)=O)ccc2)(=O)=O)C1	96.62	
RCB_C50013	10025975	NA	435.4	1-methyl-4-[5-(morpholin-4-ylmethyl)-3-furoyl]-1,4,9-triazaspiro[5.5]undecane dihydrochloride	CN(CC1)C2(CCNCC2)CN1C(c1coc(CN2CCOCC2)c1)=O	72.72	
RCB_C50024	10037740	70704262	362.5	2-({[(3-amino-2-thienyl)carbonyl]amino}methyl)-N,N-dimethyl-7,8-dihydro-4H-pyrazolo[1,5-a][1,4]diazepine-5(6H)-carboxamide	CN(C)C(N1Cc2cc(CNC(c(scc3)c3N)=O)nn2CCC1)=O	104.81	
RCB_C50027	10041741	70704267	362.5	N-cyclopropyl-N-(3-hydroxybenzyl)-2-(2,4,7-trimethyl-1H-indol-3-yl)acetamide	Cc1c(CC(N(Cc2cc(O)ccc2)C2CC2)=O)c2c(C)ccc(C)c2[nH]1	103.84	
RCB_C50029	10045111	NA	316.4	N-[(3S*,4R*)-4-ethoxytetrahydrofuran-3-yl]-N"'"-(1-phenyl-1H-pyrazol-5-yl)urea	CCO[C@@H](COC1)[C@H]1NC(Nc1ccnn1-c1ccccc1)=O	99.86	
RCB_C50030	10045854	NA	370.5	[3"'"-({4-[2-(methylthio)ethyl]piperazin-1-yl}carbonyl)biphenyl-2-yl]methanol	CSCCN(CC1)CCN1C(c1cc(-c2c(CO)cccc2)ccc1)=O	93.1	
RCB_C50033	10048011	91759616	333.4	5-({2-[2-(4-methylpiperazin-1-yl)ethyl]piperidin-1-yl}carbonyl)pyrazin-2-ol	CN1CCN(CCC(CCCC2)N2C(c(cn2)ncc2O)=O)CC1	79.25	
RCB_C50034	10049170	NA	370.5	N-(2,1,3-benzoxadiazol-5-ylmethyl)-2-[2-(2-thienyl)ethyl]piperidine-1-carboxamide	O=C(NCc1cc2nonc2cc1)N1C(CCc2cccs2)CCCC1	131.62	
RCB_C50038	10057755	70704288	374.9	N-{(3S*,4R*)-1-[(6-chloro-1,3-benzodioxol-5-yl)methyl]-4-isopropyl-3-pyrrolidinyl}methanesulfonamide	CC(C)[C@@H](CN(Cc(cc1OCOc1c1)c1Cl)C1)[C@H]1NS(C)(=O)=O	105.68	

Case 5: Commercial procurement information for a specific compound

Step 2 : Click on ID of selected entry

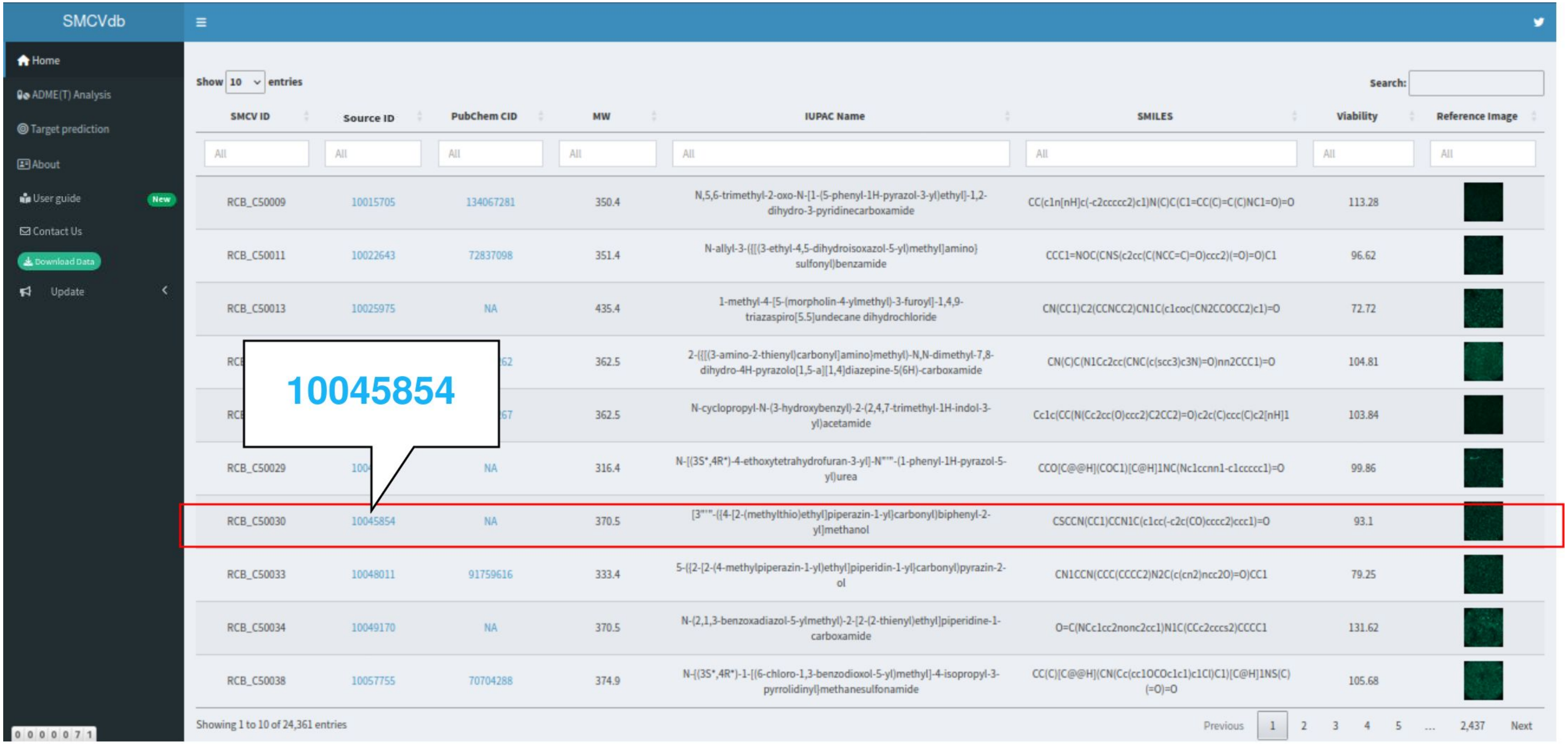

SMCV ID	Source ID	PubChem CID	MW	IUPAC Name	SMILES	Viability	Reference Image
All	All	All	All	All	All	All	All
RCB_C50009	10015705	134067281	350.4	N,5,6-trimethyl-2-oxo-N-[1-(5-phenyl-1H-pyrazol-3-yl)ethyl]-1,2-dihydro-3-pyridinecarboxamide	CC(c1n[nH]c(-c2ccccc2)c1)N(C)C(C1=CC(C)=C(C)NC1=O)=O	113.28	
RCB_C50011	10022643	72837098	351.4	N-allyl-3-({[(3-ethyl-4,5-dihydroisoxazol-5-yl)methyl]amino}sulfonyl)benzamide	CCC1=NOC(CNS(c2cc(C(NCC=C)=O)ccc2)(=O)=O)C1	96.62	
RCB_C50013	10025975	NA	435.4	1-methyl-4-[5-(morpholin-4-ylmethyl)-3-furoyl]-1,4,9-triazaspiro[5.5]undecane dihydrochloride	CN(CC1)C2(CCNCC2)CN1C(c1coc(CN2CCOCC2)c1)=O	72.72	
RC[illegible]	[illegible]	[illegible]62	362.5	2-({[(3-amino-2-thienyl)carbonyl]amino}methyl)-N,N-dimethyl-7,8-dihydro-4H-pyrazolo[1,5-a][1,4]diazepine-5(6H)-carboxamide	CN(C)C(N1Cc2cc(CNC(c(scc3)c3N)=O)nn2CCC1)=O	104.81	
RC[illegible]	[illegible]	[illegible]67	362.5	N-cyclopropyl-N-(3-hydroxybenzyl)-2-(2,4,7-trimethyl-1H-indol-3-yl)acetamide	Cc1c(CC(N(Cc2cc(O)ccc2)C2CC2)=O)c2c(C)ccc(C)c2[nH]1	103.84	
RCB_C50029	100[illegible]	NA	316.4	N-[(3S*,4R*)-4-ethoxytetrahydrofuran-3-yl]-N""-(1-phenyl-1H-pyrazol-5-yl)urea	CCO[C@@H](COC1)[C@H]1NC(Nc1ccnn1-c1ccccc1)=O	99.86	
RCB_C50030	10045854	NA	370.5	[3""-({4-[2-(methylthio)ethyl]piperazin-1-yl}carbonyl)biphenyl-2-yl]methanol	CSCCN(CC1)CCN1C(c1cc(-c2c(CO)cccc2)ccc1)=O	93.1	
RCB_C50033	10048011	91759616	333.4	5-({2-[2-(4-methylpiperazin-1-yl)ethyl]piperidin-1-yl}carbonyl)pyrazin-2-ol	CN1CCN(CCC(CCCC2)N2C(c(cn2)ncc2O)=O)CC1	79.25	
RCB_C50034	10049170	NA	370.5	N-(2,1,3-benzoxadiazol-5-ylmethyl)-2-[2-(2-thienyl)ethyl]piperidine-1-carboxamide	O=C(NCc1cc2nonc2cc1)N1C(CCc2cccs2)CCCC1	131.62	
RCB_C50038	10057755	70704288	374.9	N-{(3S*,4R*)-1-[(6-chloro-1,3-benzodioxol-5-yl)methyl]-4-isopropyl-3-pyrrolidinyl}methanesulfonamide	CC(C)[C@@H](CN(Cc(cc1OCOc1c1)c1Cl)C1)[C@H]1NS(C)(=O)=O	105.68	

Case 5: Commercial procurement information for a specific compound

Step 3 : The source page will appear, Follow the intsructions provided by vendor to order

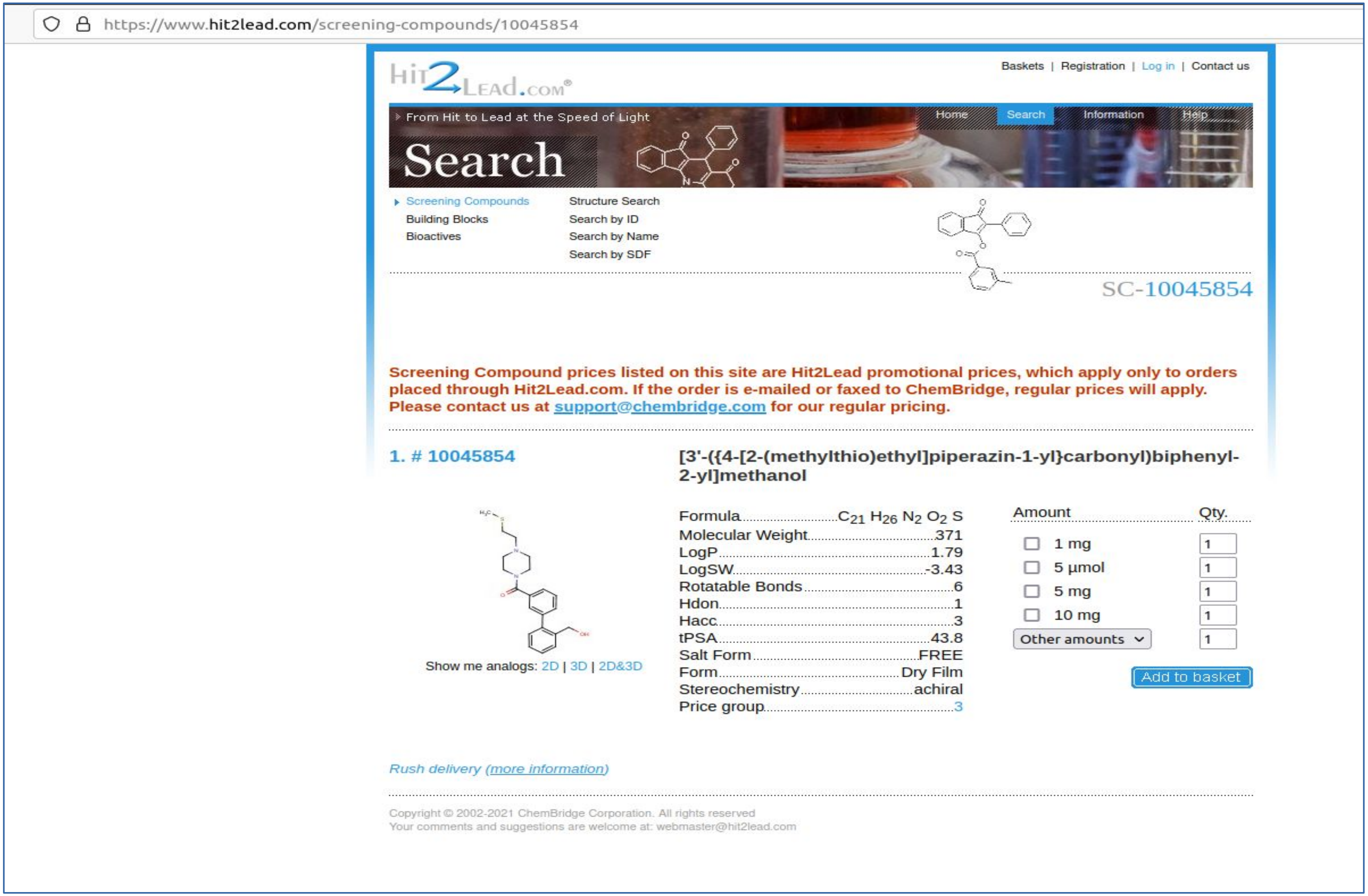

Case 6: Downloading compound in molecular docking-supported format

Step 1 : Choose your compound using case 1-3

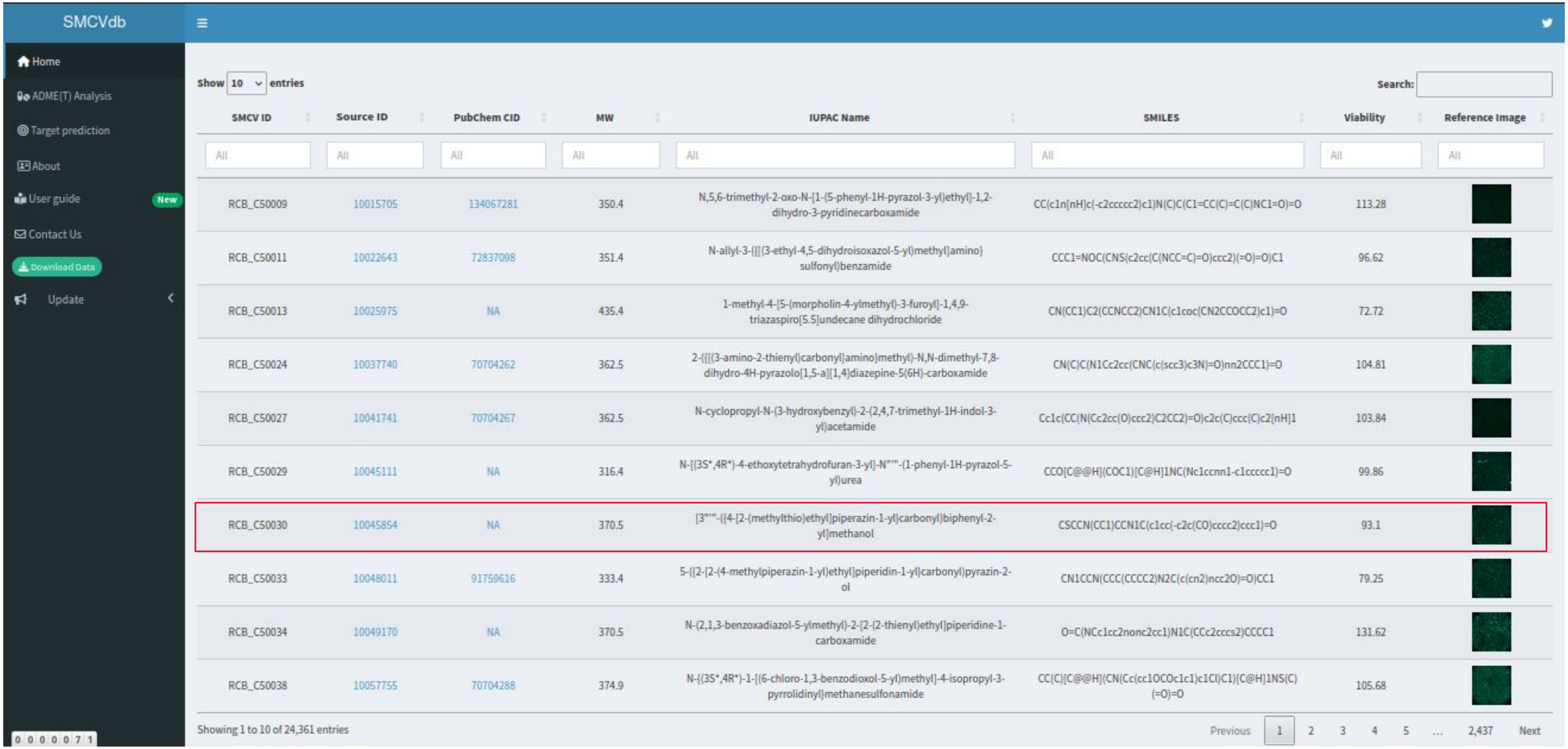

SMCV ID	Source ID	PubChem CID	MW	IUPAC Name	SMILES	Viability	Reference Image
RCB_C50009	10015705	134067281	350.4	N,5,6-trimethyl-2-oxo-N-[1-(5-phenyl-1H-pyrazol-3-yl)ethyl]-1,2-dihydro-3-pyridinecarboxamide	CC(c1n[nH]c(-c2ccccc2)c1)N(C)C(C1=CC(C)=C(C)NC1=O)=O	113.28	
RCB_C50011	10022643	72837098	351.4	N-allyl-3-({[(3-ethyl-4,5-dihydroisoxazol-5-yl)methyl]amino}sulfonyl)benzamide	CCC1=NOC(CNS(c2cc(C(NCC=C)=O)ccc2)(=O)=O)C1	96.62	
RCB_C50013	10025975	NA	435.4	1-methyl-4-[5-(morpholin-4-ylmethyl)-3-furoyl]-1,4,9-triazaspiro[5.5]undecane dihydrochloride	CN(CC1)C2(CCNCC2)CN1C(c1coc(CN2CCOCC2)c1)=O	72.72	
RCB_C50024	10037740	70704262	362.5	2-({[(3-amino-2-thienyl)carbonyl]amino}methyl)-N,N-dimethyl-7,8-dihydro-4H-pyrazolo[1,5-a][1,4]diazepine-5(6H)-carboxamide	CN(C)C(N1Cc2cc(CNC(c(scc3)c3N)=O)nn2CCC1)=O	104.81	
RCB_C50027	10041741	70704267	362.5	N-cyclopropyl-N-(3-hydroxybenzyl)-2-(2,4,7-trimethyl-1H-indol-3-yl)acetamide	Cc1c(CC(N(Cc2cc(O)ccc2)C2CC2)=O)c2c(C)ccc(C)c2[nH]1	103.84	
RCB_C50029	10045111	NA	316.4	N-[(3S*,4R*)-4-ethoxytetrahydrofuran-3-yl]-N''''-(1-phenyl-1H-pyrazol-5-yl)urea	CCO[C@@H](COC1)[C@H]1NC(Nc1ccnn1-c1ccccc1)=O	99.86	
RCB_C50030	10045854	NA	370.5	[3''''-({4-[2-(methylthio)ethyl]piperazin-1-yl}carbonyl)biphenyl-2-yl]methanol	CSCCN(CC1)CCN1C(c1cc(-c2c(CO)cccc2)ccc1)=O	93.1	
RCB_C50033	10048011	91759616	333.4	5-({2-[2-(4-methylpiperazin-1-yl)ethyl]piperidin-1-yl}carbonyl)pyrazin-2-ol	CN1CCN(CCC(CCCC2)N2C(c(cn2)ncc2O)=O)CC1	79.25	
RCB_C50034	10049170	NA	370.5	N-(2,1,3-benzoxadiazol-5-ylmethyl)-2-[2-(2-thienyl)ethyl]piperidine-1-carboxamide	O=C(NCc1cc2nonc2cc1)N1C(CCc2cccs2)CCCC1	131.62	
RCB_C50038	10057755	70704288	374.9	N-{(3S*,4R*)-1-[(6-chloro-1,3-benzodioxol-5-yl)methyl]-4-isopropyl-3-pyrrolidinyl}methanesulfonamide	CC(C)[C@@H](CN(Cc(cc1OCOc1c1)c1Cl)C1)[C@H]1NS(C)(=O)=O	105.68	

Case 6: Downloading compound in molecular docking-supported format

Step 2 : Click on ID of selected entry

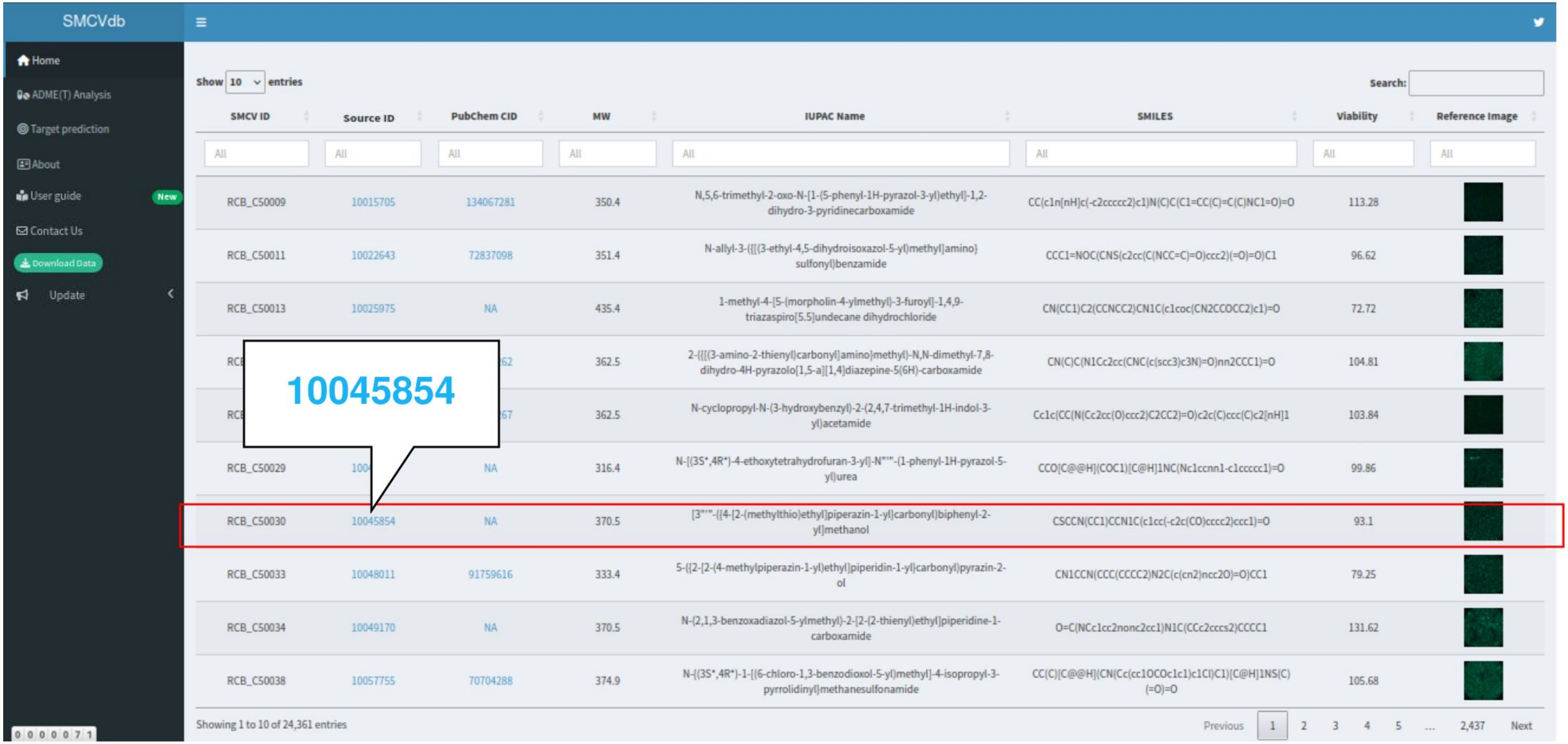

Case 6: Downloading compound in molecular docking-supported format

Step 3 : The source page will appear

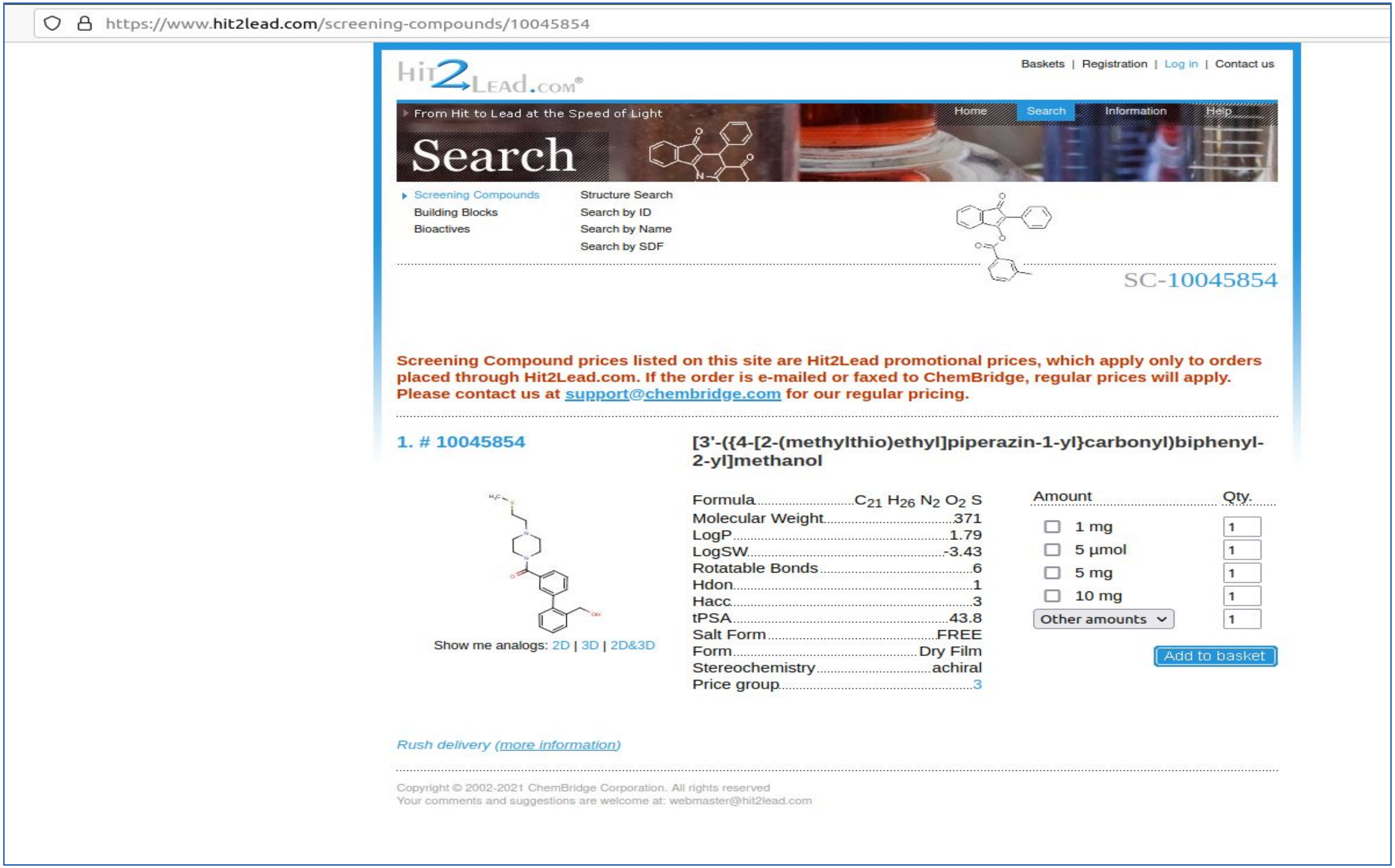

Source : www.hit2lead.com/

Case 6: Downloading compound in molecular docking-supported format

Step 4 : click on image

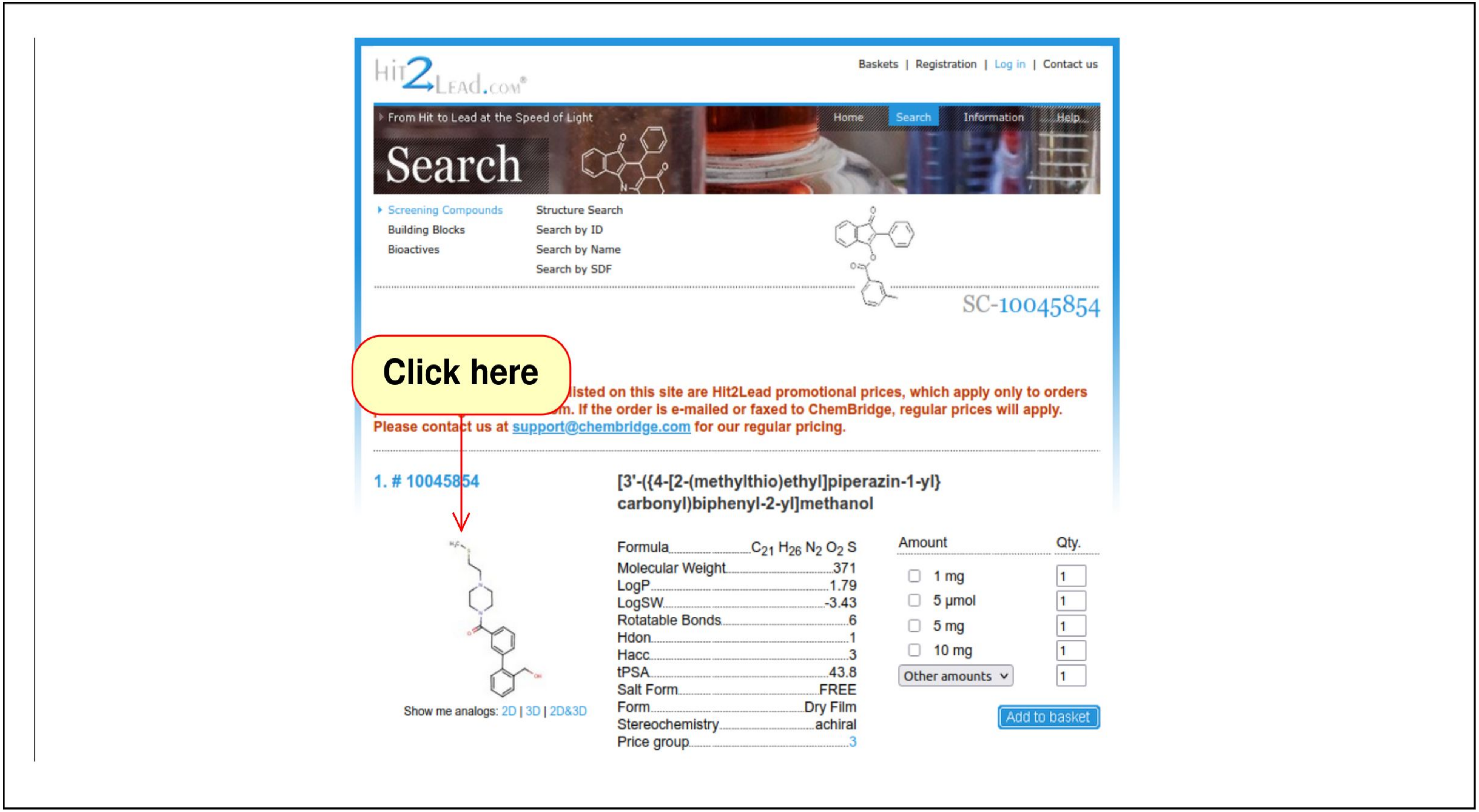

Source : www.hit2lead.com/

Case 6: Downloading compound in molecular docking-supported format

Step 5 : Click save icon (💾)

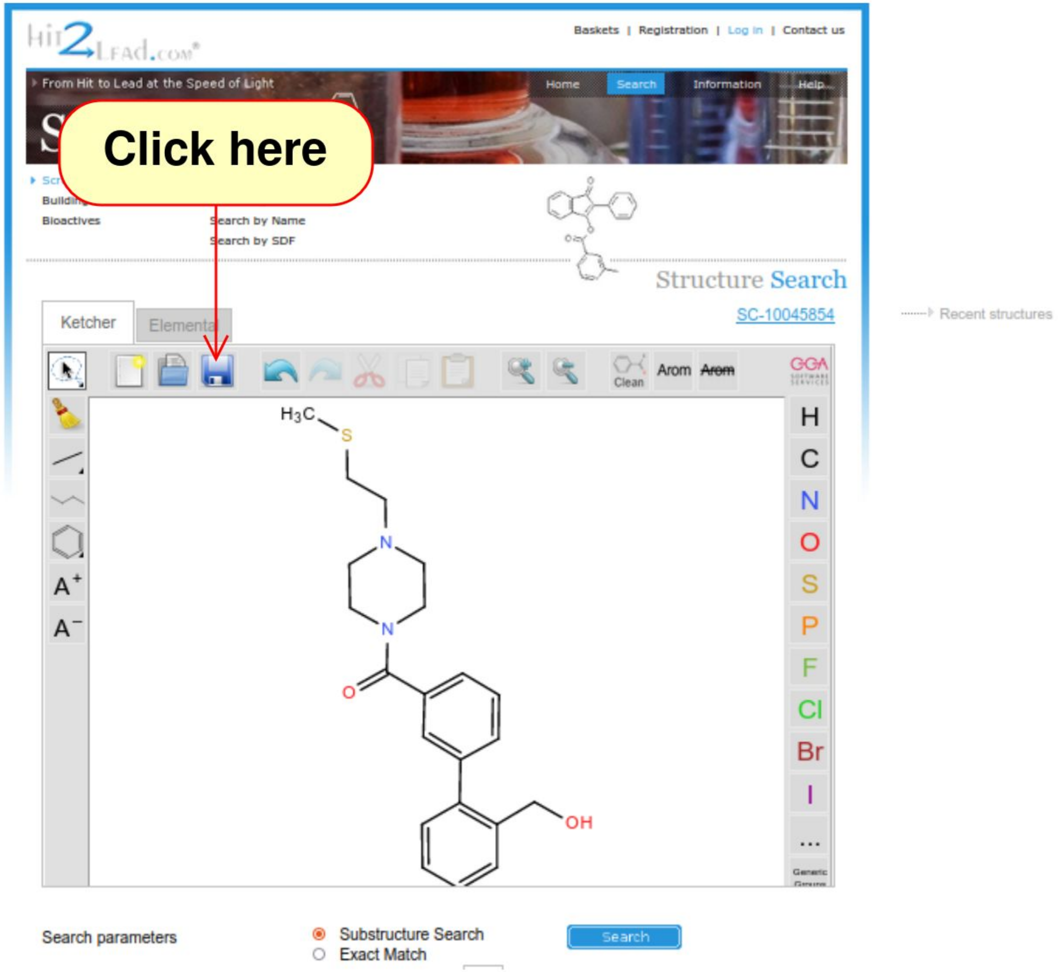

Source : www.hit2lead.com/

Case 6: Downloading compound in molecular docking-supported format

Step 6 : save it in MOL format.

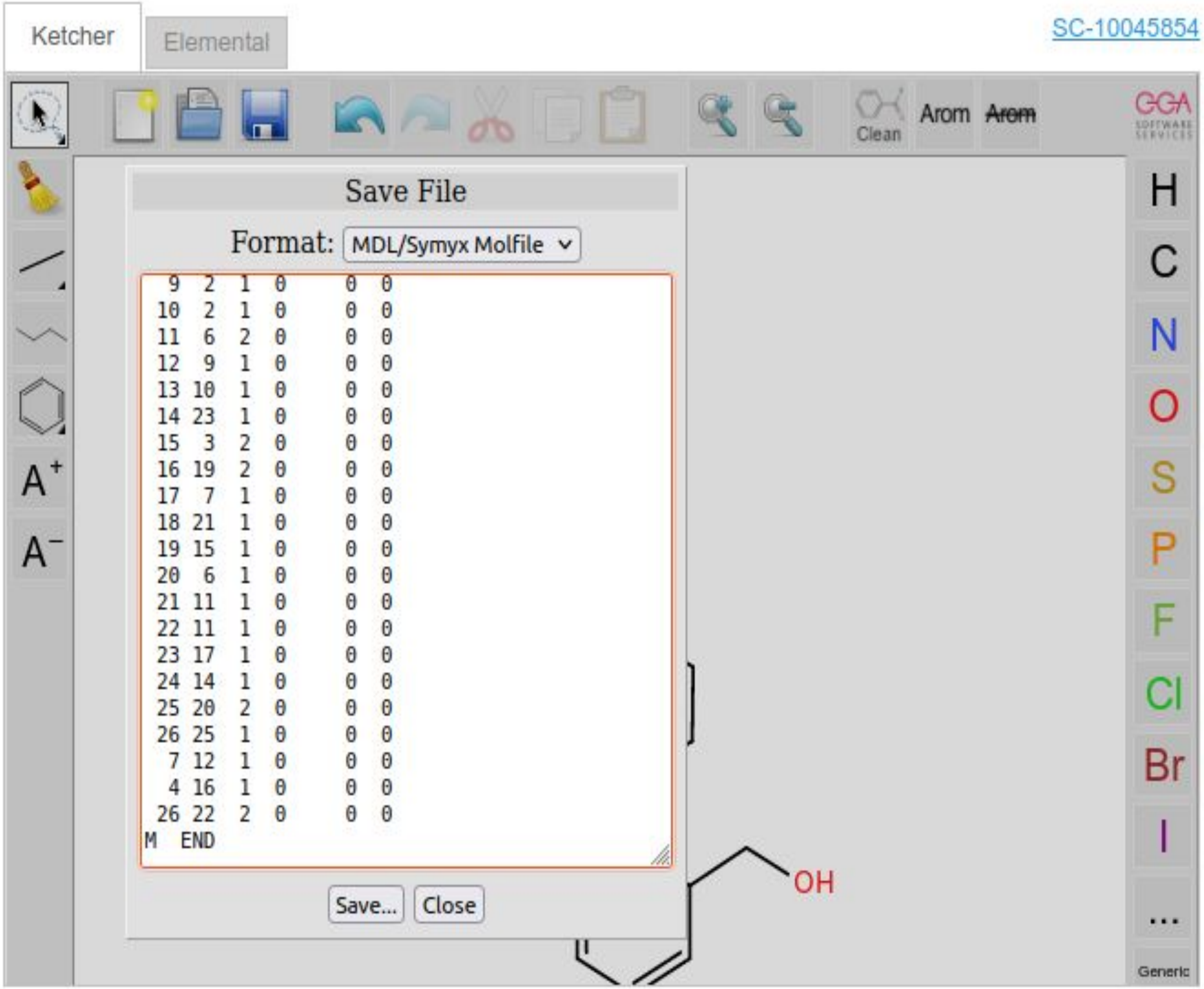

NOTE : MOL format is acceptable for molecular docking tools

Source : www.hit2lead.com/

Tutorial main

Case 7: Accessing PubChem Information for a specific compound

Step 1 : Choose your compound using case 1-3, then go to “PubChem CID”

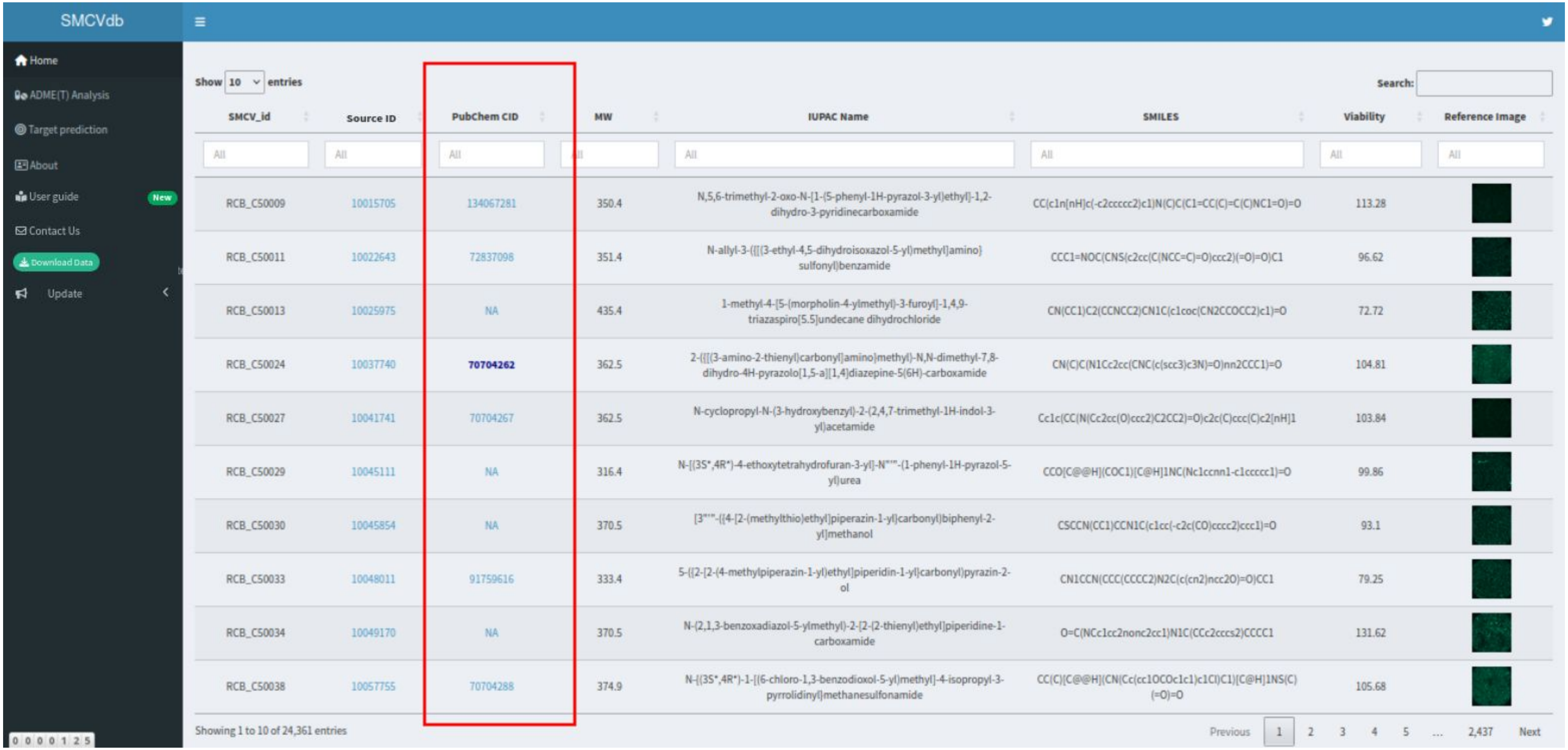

SMCV_id	Source ID	PubChem CID	MW	IUPAC Name	SMILES	Viability	Reference Image
All	All	All	All	All	All	All	All
RCB_C50009	10015705	134067281	350.4	N,5,6-trimethyl-2-oxo-N-[1-(5-phenyl-1H-pyrazol-3-yl)ethyl]-1,2-dihydro-3-pyridinecarboxamide	CC(c1n[nH]c(-c2ccccc2)c1)N(C)C(C1=CC(C)=C(C)NC1=O)=O	113.28	
RCB_C50011	10022643	72837098	351.4	N-allyl-3-({[(3-ethyl-4,5-dihydroisoxazol-5-yl)methyl]amino}sulfonyl)benzamide	CCC1=NOC(CNS(c2cc(C(NCC=C)=O)ccc2)(=O)=O)C1	96.62	
RCB_C50013	10025975	NA	435.4	1-methyl-4-[5-(morpholin-4-ylmethyl)-3-furoyl]-1,4,9-triazaspiro[5.5]undecane dihydrochloride	CN(CC1)C2(CCNCC2)CN1C(c1coc(CN2CCOCC2)c1)=O	72.72	
RCB_C50024	10037740	**70704262**	362.5	2-({[(3-amino-2-thienyl)carbonyl]amino}methyl)-N,N-dimethyl-7,8-dihydro-4H-pyrazolo[1,5-a][1,4]diazepine-5(6H)-carboxamide	CN(C)C(N1Cc2cc(CNC(c(scc3)c3N)=O)nn2CCC1)=O	104.81	
RCB_C50027	10041741	70704267	362.5	N-cyclopropyl-N-(3-hydroxybenzyl)-2-(2,4,7-trimethyl-1H-indol-3-yl)acetamide	Cc1c(CC(N(Cc2cc(O)ccc2)C2CC2)=O)c2c(C)ccc(C)c2[nH]1	103.84	
RCB_C50029	10045111	NA	316.4	N-[(3S*,4R*)-4-ethoxytetrahydrofuran-3-yl]-N''''-(1-phenyl-1H-pyrazol-5-yl)urea	CCO[C@@H](COC1)[C@H]1NC(Nc1ccnn1-c1ccccc1)=O	99.86	
RCB_C50030	10045854	NA	370.5	[3''''-({4-[2-(methylthio)ethyl]piperazin-1-yl}carbonyl)biphenyl-2-yl]methanol	CSCCN(CC1)CCN1C(c1cc(-c2c(CO)cccc2)ccc1)=O	93.1	
RCB_C50033	10048011	91759616	333.4	5-({2-[2-(4-methylpiperazin-1-yl)ethyl]piperidin-1-yl}carbonyl)pyrazin-2-ol	CN1CCN(CCC(CCCC2)N2C(c(cn2)ncc2O)=O)CC1	79.25	
RCB_C50034	10049170	NA	370.5	N-(2,1,3-benzoxadiazol-5-ylmethyl)-2-[2-(2-thienyl)ethyl]piperidine-1-carboxamide	O=C(NCc1cc2nonc2cc1)N1C(CCc2cccs2)CCCC1	131.62	
RCB_C50038	10057755	70704288	374.9	N-{(3S*,4R*)-1-[(6-chloro-1,3-benzodioxol-5-yl)methyl]-4-isopropyl-3-pyrrolidinyl}methanesulfonamide	CC(C)[C@@H](CN(Cc(cc1OCOc1c1)c1Cl)C1)[C@H]1NS(C)(=O)=O	105.68	

Showing 1 to 10 of 24,361 entries

Previous 1 2 3 4 5 ... 2,437 Next

Note : Compounds with available cytotoxicity data in PubChem are Highlighted in navy

Case 7: Accessing PubChem Information for a specific compound

Step 2 : Click the link

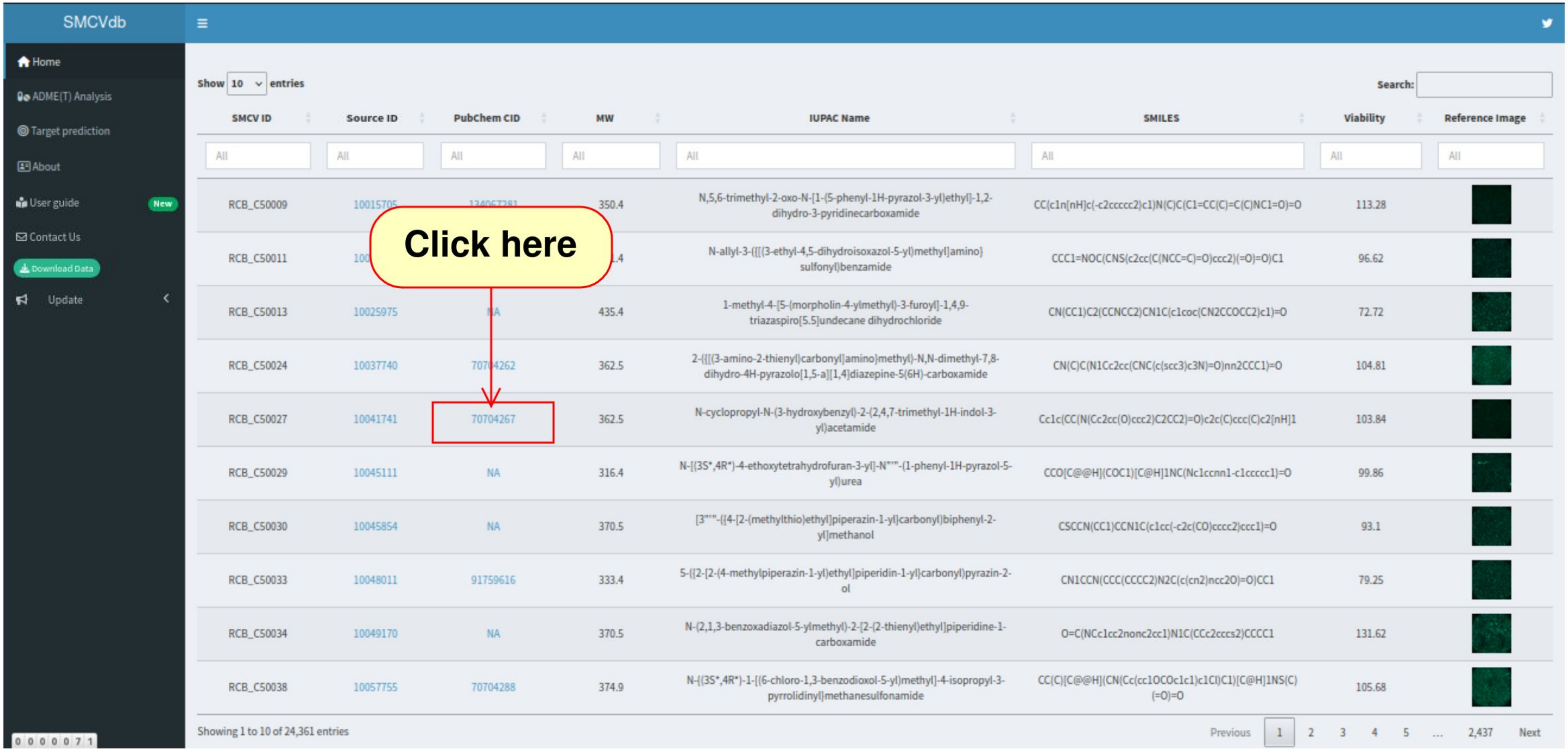

Case 7: Accessing PubChem Information for a specific compound

Step 3 : The Pubchem page assosiated with selected name will appear

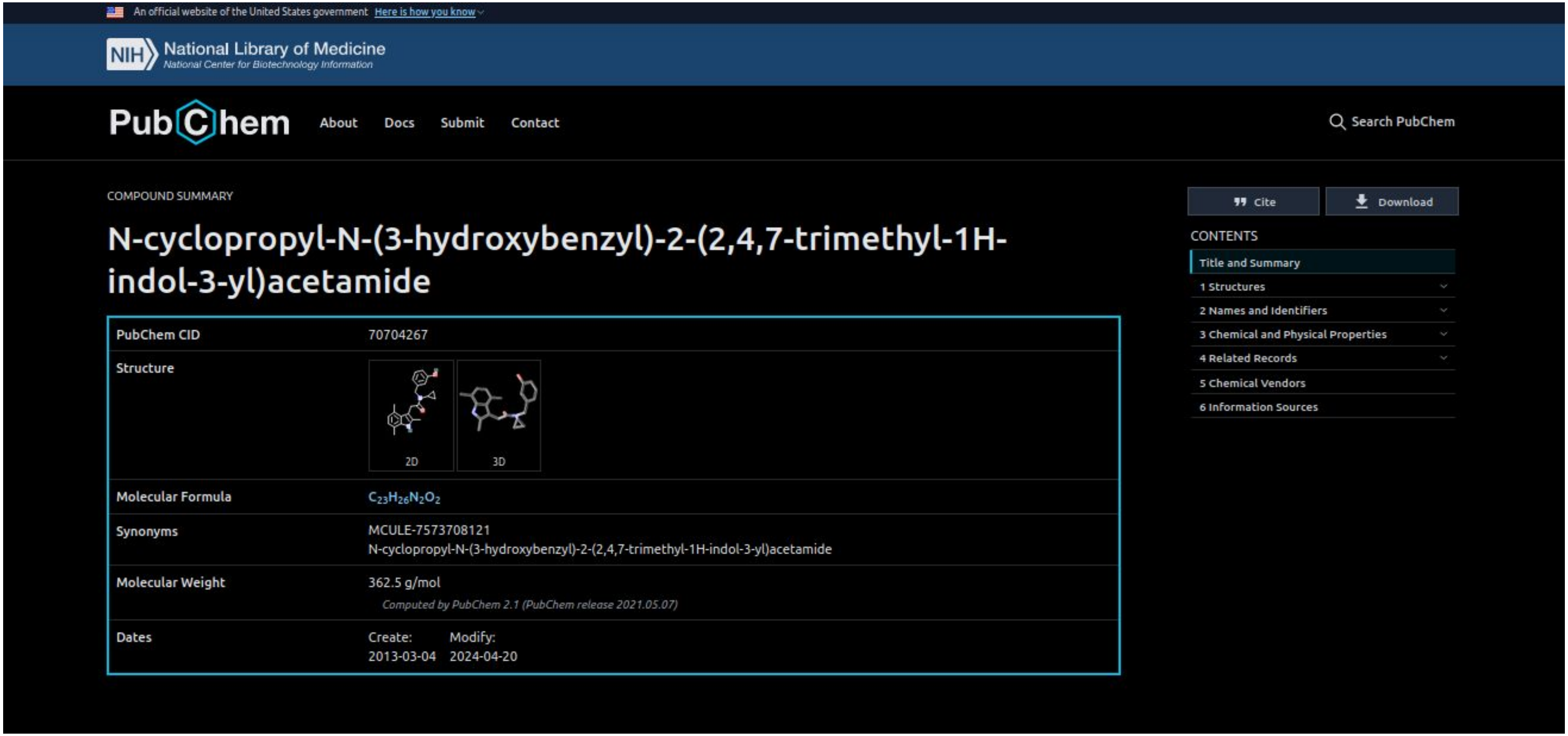

Source : https://pubchem.ncbi.nlm.nih.gov/

Case 8: Performing ADME(T) analysis of a selected compound

Step 1 : Choose your compound using case 1-3 and copy the smile notation

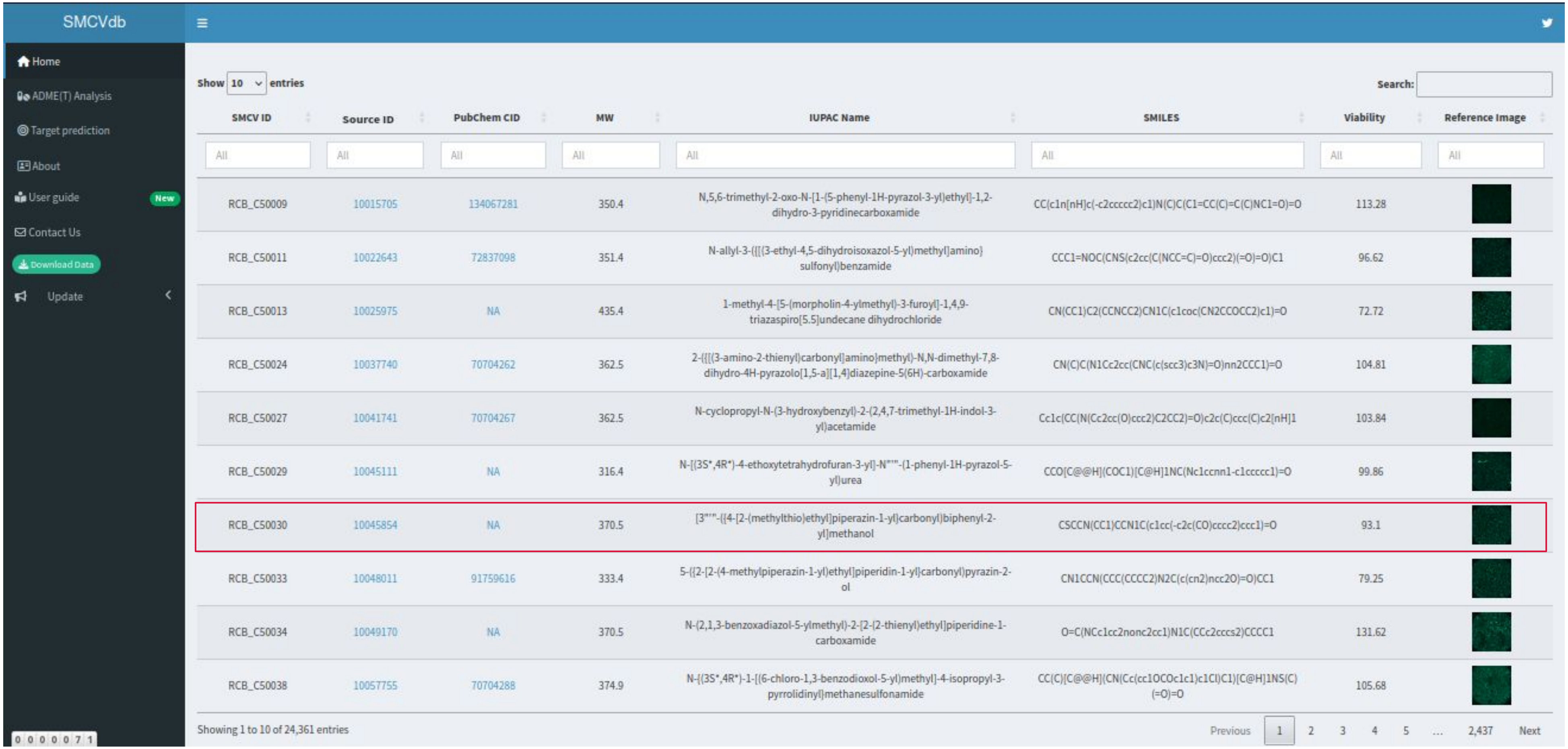

SMCV ID	Source ID	PubChem CID	MW	IUPAC Name	SMILES	Viability	Reference Image
RCB_C50009	10015705	134067281	350.4	N,5,6-trimethyl-2-oxo-N-[1-(5-phenyl-1H-pyrazol-3-yl)ethyl]-1,2-dihydro-3-pyridinecarboxamide	CC(c1n[nH]c(-c2ccccc2)c1)N(C)C(C1=CC(C)=C(C)NC1=O)=O	113.28	
RCB_C50011	10022643	72837098	351.4	N-allyl-3-({[(3-ethyl-4,5-dihydroisoxazol-5-yl)methyl]amino}sulfonyl)benzamide	CCC1=NOC(CNS(c2cc(C(NCC=C)=O)ccc2)(=O)=O)C1	96.62	
RCB_C50013	10025975	NA	435.4	1-methyl-4-[5-(morpholin-4-ylmethyl)-3-furoyl]-1,4,9-triazaspiro[5.5]undecane dihydrochloride	CN(CC1)C2(CCNCC2)CN1C(c1coc(CN2CCOCC2)c1)=O	72.72	
RCB_C50024	10037740	70704262	362.5	2-({[(3-amino-2-thienyl)carbonyl]amino}methyl)-N,N-dimethyl-7,8-dihydro-4H-pyrazolo[1,5-a][1,4]diazepine-5(6H)-carboxamide	CN(C)C(N1Cc2cc(CNC(c(scc3)c3N)=O)nn2CCC1)=O	104.81	
RCB_C50027	10041741	70704267	362.5	N-cyclopropyl-N-(3-hydroxybenzyl)-2-(2,4,7-trimethyl-1H-indol-3-yl)acetamide	Cc1c(CC(N(Cc2cc(O)ccc2)C2CC2)=O)c2c(C)ccc(C)c2[nH]1	103.84	
RCB_C50029	10045111	NA	316.4	N-[(3S*,4R*)-4-ethoxytetrahydrofuran-3-yl]-N"'"-(1-phenyl-1H-pyrazol-5-yl)urea	CCO[C@@H](COC1)[C@H]1NC(Nc1ccnn1-c1ccccc1)=O	99.86	
RCB_C50030	10045854	NA	370.5	[3"'"-({4-[2-(methylthio)ethyl]piperazin-1-yl}carbonyl)biphenyl-2-yl]methanol	CSCCN(CC1)CCN1C(c1cc(-c2c(CO)cccc2)ccc1)=O	93.1	
RCB_C50033	10048011	91759616	333.4	5-({2-[2-(4-methylpiperazin-1-yl)ethyl]piperidin-1-yl}carbonyl)pyrazin-2-ol	CN1CCN(CCC(CCCC2)N2C(c(cn2)ncc2O)=O)CC1	79.25	
RCB_C50034	10049170	NA	370.5	N-(2,1,3-benzoxadiazol-5-ylmethyl)-2-[2-(2-thienyl)ethyl]piperidine-1-carboxamide	O=C(NCc1cc2nonc2cc1)N1C(CCc2cccs2)CCCC1	131.62	
RCB_C50038	10057755	70704288	374.9	N-{(3S*,4R*)-1-[(6-chloro-1,3-benzodioxol-5-yl)methyl]-4-isopropyl-3-pyrrolidinyl}methanesulfonamide	CC(C)[C@@H](CN(Cc(cc1OCOc1c1)c1Cl)C1)[C@H]1NS(C)(=O)=O	105.68	

Showing 1 to 10 of 24,361 entries

Previous 1 2 3 4 5 … 2,437 Next

Case 8: Performing ADME(T) analysis of a selected compound

Step 2 : Click on 'ADME(T) Analysis' from side panel

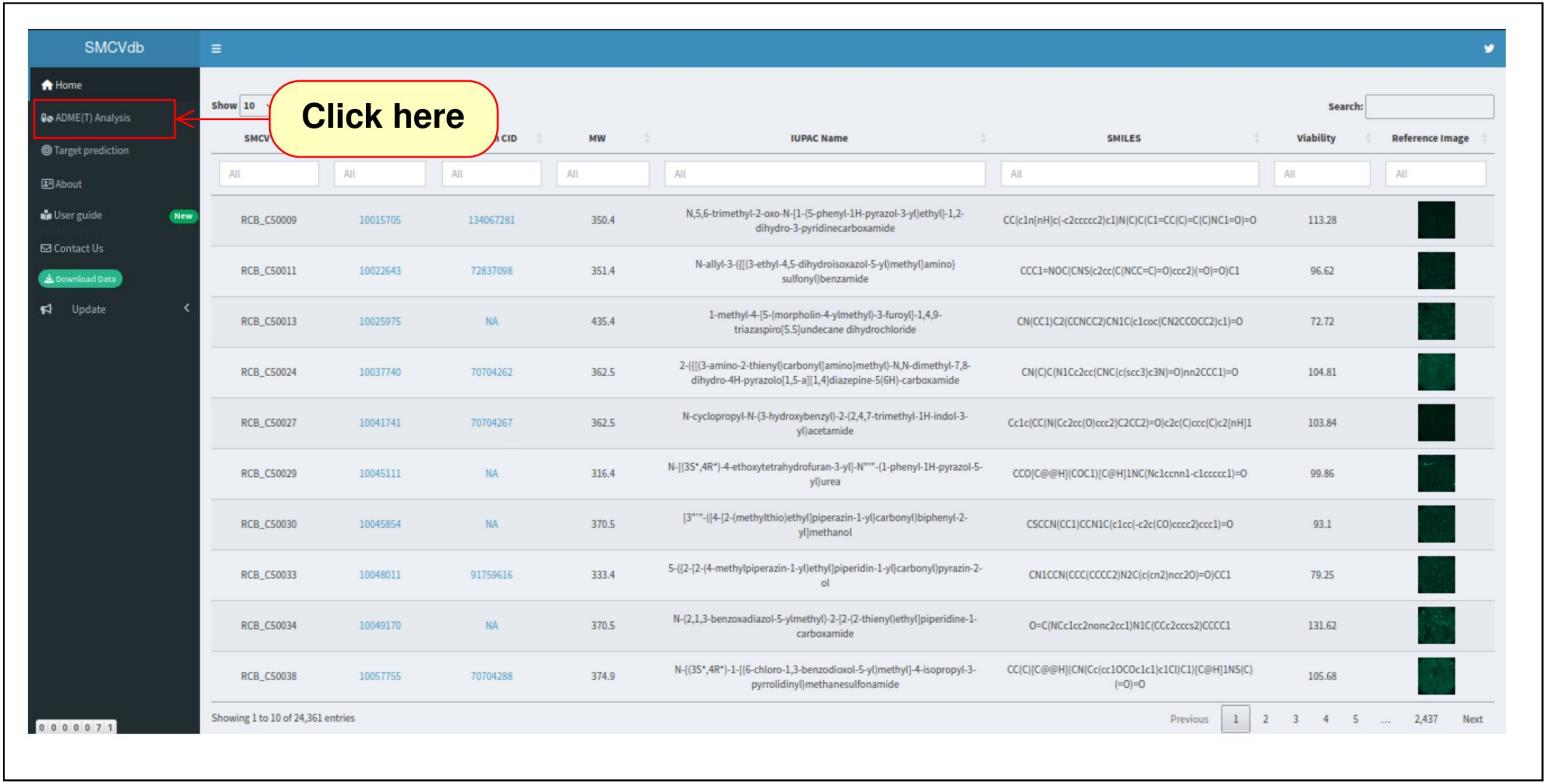

Case 8: Performing ADME(T) analysis of a selected compound

Step 3 : Click on the server link eg: SwissADME

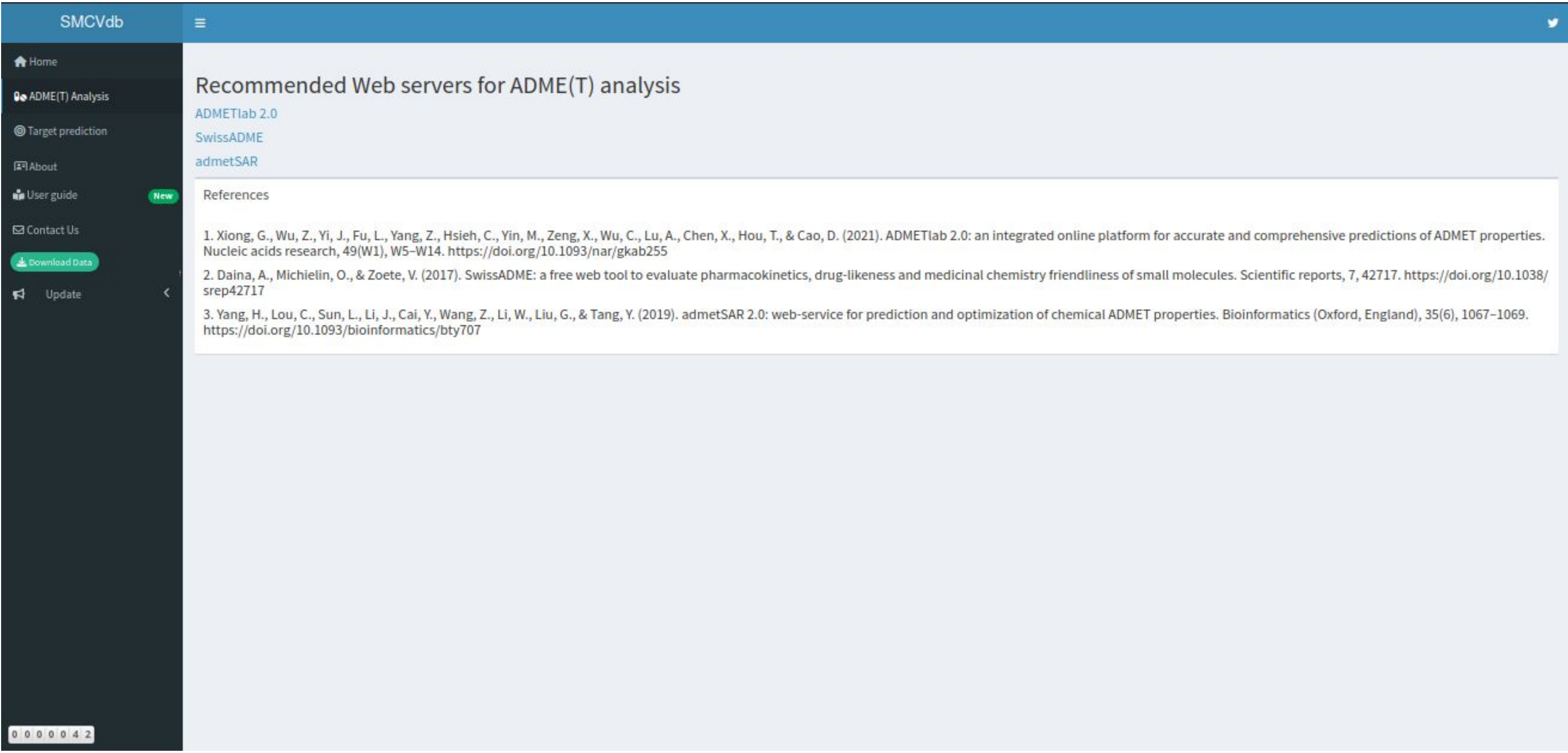

Case 8: Performing ADME(T) analysis of a selected compound

Step 4 : Paste the SMILES notation and click RUN

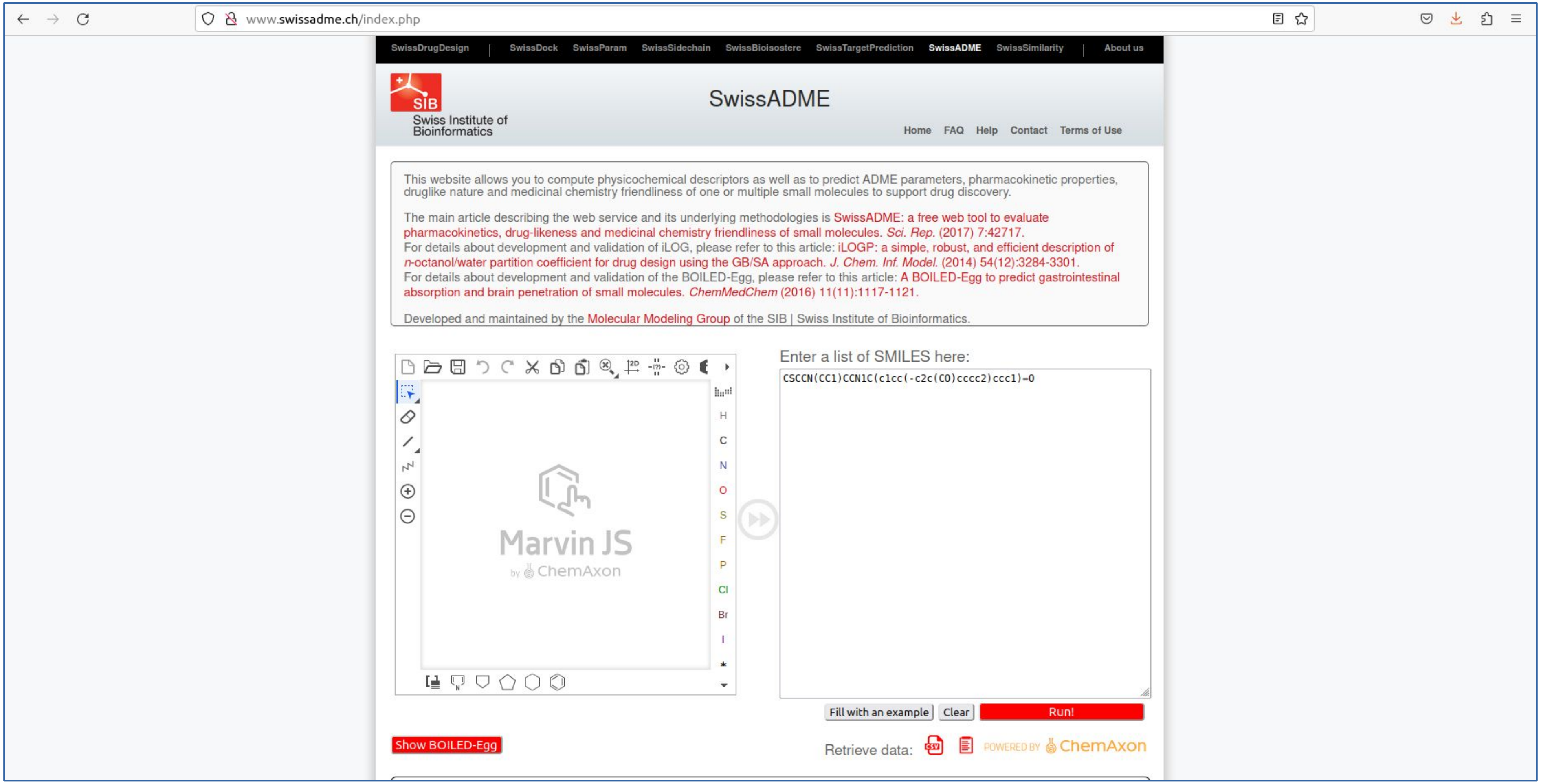

Source : http://www.swissadme.ch/

Case 8: Performing ADME(T) analysis of a selected compound

Step 5 : Result page will appear

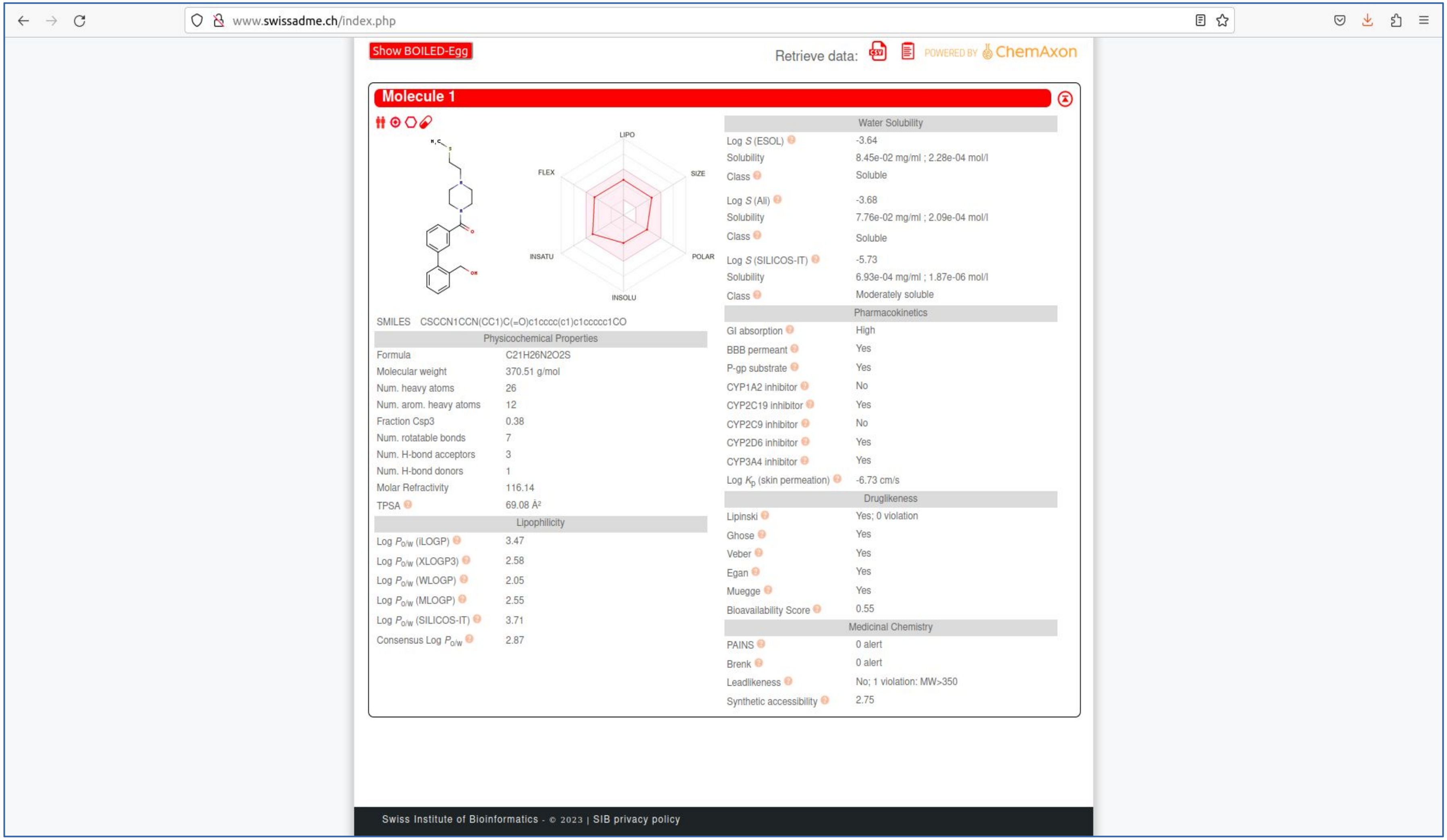

Source : http://www.swissadme.ch/

Case 9: Target prediction for a specific compound

Tutorial main

Step 1 : Choose your compound using case 1-3 and copy the smile notation

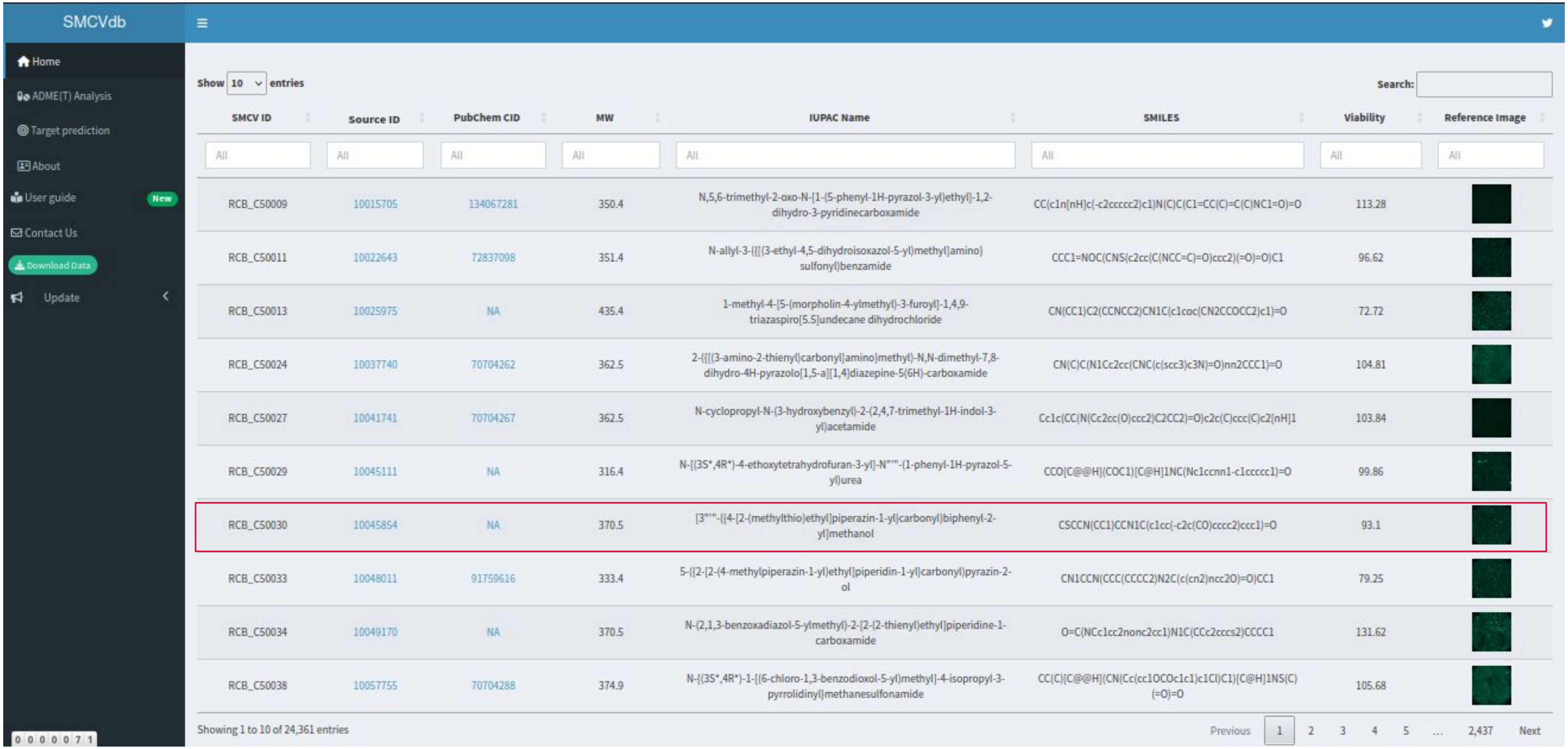

SMCV ID	Source ID	PubChem CID	MW	IUPAC Name	SMILES	Viability	Reference Image
All	All	All	All	All	All	All	All
RCB_C50009	10015705	134067281	350.4	N,5,6-trimethyl-2-oxo-N-[1-(5-phenyl-1H-pyrazol-3-yl)ethyl]-1,2-dihydro-3-pyridinecarboxamide	CC(c1n[nH]c(-c2ccccc2)c1)N(C)C(C1=CC(C)=C(C)NC1=O)=O	113.28	
RCB_C50011	10022643	72837098	351.4	N-allyl-3-({[(3-ethyl-4,5-dihydroisoxazol-5-yl)methyl]amino}sulfonyl)benzamide	CCC1=NOC(CNS(c2cc(C(NCC=C)=O)ccc2)(=O)=O)C1	96.62	
RCB_C50013	10025975	NA	435.4	1-methyl-4-[5-(morpholin-4-ylmethyl)-3-furoyl]-1,4,9-triazaspiro[5.5]undecane dihydrochloride	CN(CC1)C2(CCNCC2)CN1C(c1coc(CN2CCOCC2)c1)=O	72.72	
RCB_C50024	10037740	70704262	362.5	2-({[(3-amino-2-thienyl)carbonyl]amino}methyl)-N,N-dimethyl-7,8-dihydro-4H-pyrazolo[1,5-a][1,4]diazepine-5(6H)-carboxamide	CN(C)C(N1Cc2cc(CNC(c(scc3)c3N)=O)nn2CCC1)=O	104.81	
RCB_C50027	10041741	70704267	362.5	N-cyclopropyl-N-(3-hydroxybenzyl)-2-(2,4,7-trimethyl-1H-indol-3-yl)acetamide	Cc1c(CC(N(Cc2cc(O)ccc2)C2CC2)=O)c2c(C)ccc(C)c2[nH]1	103.84	
RCB_C50029	10045111	NA	316.4	N-[(3S*,4R*)-4-ethoxytetrahydrofuran-3-yl]-N"'"-(1-phenyl-1H-pyrazol-5-yl)urea	CCO[C@@H](COC1)[C@H]1NC(Nc1ccnn1-c1ccccc1)=O	99.86	
RCB_C50030	10045854	NA	370.5	[3"'"-({4-[2-(methylthio)ethyl]piperazin-1-yl}carbonyl)biphenyl-2-yl]methanol	CSCCN(CC1)CCN1C(c1cc(-c2c(CO)cccc2)ccc1)=O	93.1	
RCB_C50033	10048011	91759616	333.4	5-({2-[2-(4-methylpiperazin-1-yl)ethyl]piperidin-1-yl}carbonyl)pyrazin-2-ol	CN1CCN(CCC(CCCC2)N2C(c(cn2)ncc2O)=O)CC1	79.25	
RCB_C50034	10049170	NA	370.5	N-(2,1,3-benzoxadiazol-5-ylmethyl)-2-[2-(2-thienyl)ethyl]piperidine-1-carboxamide	O=C(NCc1cc2nonc2cc1)N1C(CCc2cccs2)CCCC1	131.62	
RCB_C50038	10057755	70704288	374.9	N-{(3S*,4R*)-1-[(6-chloro-1,3-benzodioxol-5-yl)methyl]-4-isopropyl-3-pyrrolidinyl}methanesulfonamide	CC(C)[C@@H](CN(Cc(cc1OCOc1c1)c1Cl)C1)[C@H]1NS(C)(=O)=O	105.68	

Showing 1 to 10 of 24,361 entries

Previous 1 2 3 4 5 … 2,437 Next

Case 9: Target prediction for a specific compound

Tutorial main

Step 2 : Click on 'Target prediction' from side panel

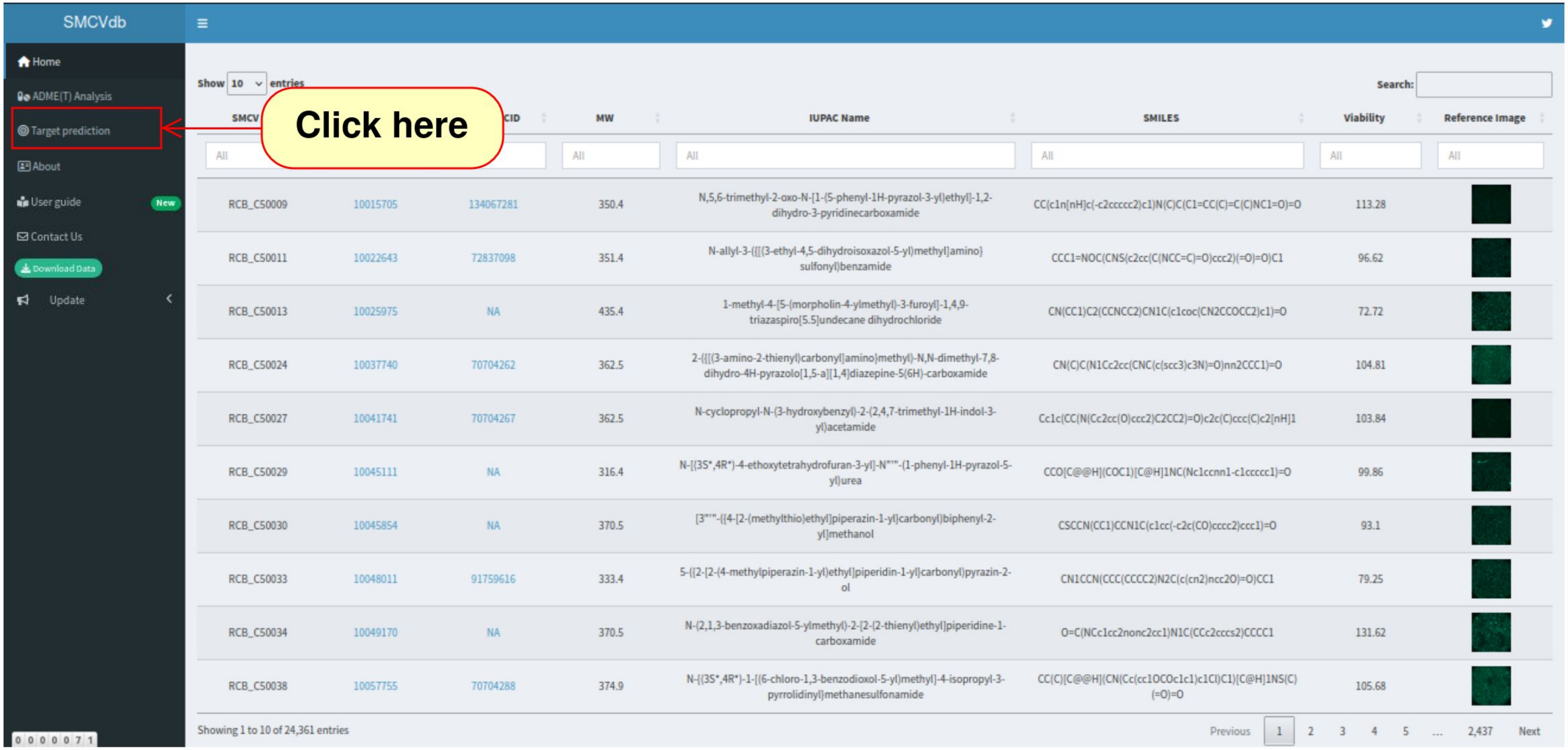

SMCV		CID	MW	IUPAC Name	SMILES	Viability	Reference Image
All			All	All	All	All	All
RCB_C50009	10015705	134067281	350.4	N,5,6-trimethyl-2-oxo-N-[1-(5-phenyl-1H-pyrazol-3-yl)ethyl]-1,2-dihydro-3-pyridinecarboxamide	CC(c1n[nH]c(-c2ccccc2)c1)N(C)C(C1=CC(C)=C(C)NC1=O)=O	113.28	
RCB_C50011	10022643	72837098	351.4	N-allyl-3-({[(3-ethyl-4,5-dihydroisoxazol-5-yl)methyl]amino}sulfonyl)benzamide	CCC1=NOC(CNS(c2cc(C(NCC=C)=O)ccc2)(=O)=O)C1	96.62	
RCB_C50013	10025975	NA	435.4	1-methyl-4-[5-(morpholin-4-ylmethyl)-3-furoyl]-1,4,9-triazaspiro[5.5]undecane dihydrochloride	CN(CC1)C2(CCNCC2)CN1C(c1coc(CN2CCOCC2)c1)=O	72.72	
RCB_C50024	10037740	70704262	362.5	2-({[(3-amino-2-thienyl)carbonyl]amino}methyl)-N,N-dimethyl-7,8-dihydro-4H-pyrazolo[1,5-a][1,4]diazepine-5(6H)-carboxamide	CN(C)C(N1Cc2cc(CNC(c(scc3)c3N)=O)nn2CCC1)=O	104.81	
RCB_C50027	10041741	70704267	362.5	N-cyclopropyl-N-(3-hydroxybenzyl)-2-(2,4,7-trimethyl-1H-indol-3-yl)acetamide	Cc1c(CC(N(Cc2cc(O)ccc2)C2CC2)=O)c2c(C)ccc(C)c2[nH]1	103.84	
RCB_C50029	10045111	NA	316.4	N-[(3S*,4R*)-4-ethoxytetrahydrofuran-3-yl]-N"'"-(1-phenyl-1H-pyrazol-5-yl)urea	CCO[C@@H](COC1)[C@H]1NC(Nc1ccnn1-c1ccccc1)=O	99.86	
RCB_C50030	10045854	NA	370.5	[3"'"-({4-[2-(methylthio)ethyl]piperazin-1-yl}carbonyl)biphenyl-2-yl]methanol	CSCCN(CC1)CCN1C(c1cc(-c2c(CO)cccc2)ccc1)=O	93.1	
RCB_C50033	10048011	91759616	333.4	5-({2-[2-(4-methylpiperazin-1-yl)ethyl]piperidin-1-yl}carbonyl)pyrazin-2-ol	CN1CCN(CCC(CCCC2)N2C(c(cn2)ncc2O)=O)CC1	79.25	
RCB_C50034	10049170	NA	370.5	N-(2,1,3-benzoxadiazol-5-ylmethyl)-2-[2-(2-thienyl)ethyl]piperidine-1-carboxamide	O=C(NCc1cc2nonc2cc1)N1C(CCc2cccs2)CCCC1	131.62	
RCB_C50038	10057755	70704288	374.9	N-{(3S*,4R*)-1-[(6-chloro-1,3-benzodioxol-5-yl)methyl]-4-isopropyl-3-pyrrolidinyl}methanesulfonamide	CC(C)[C@@H](CN(Cc(cc1OCOc1c1)c1Cl)C1)[C@H]1NS(C)(=O)=O	105.68	

Showing 1 to 10 of 24,361 entries

Case 9: Target prediction for a specific compound

Tutorial main

Step 3 : Click on SwissTargetPrediction

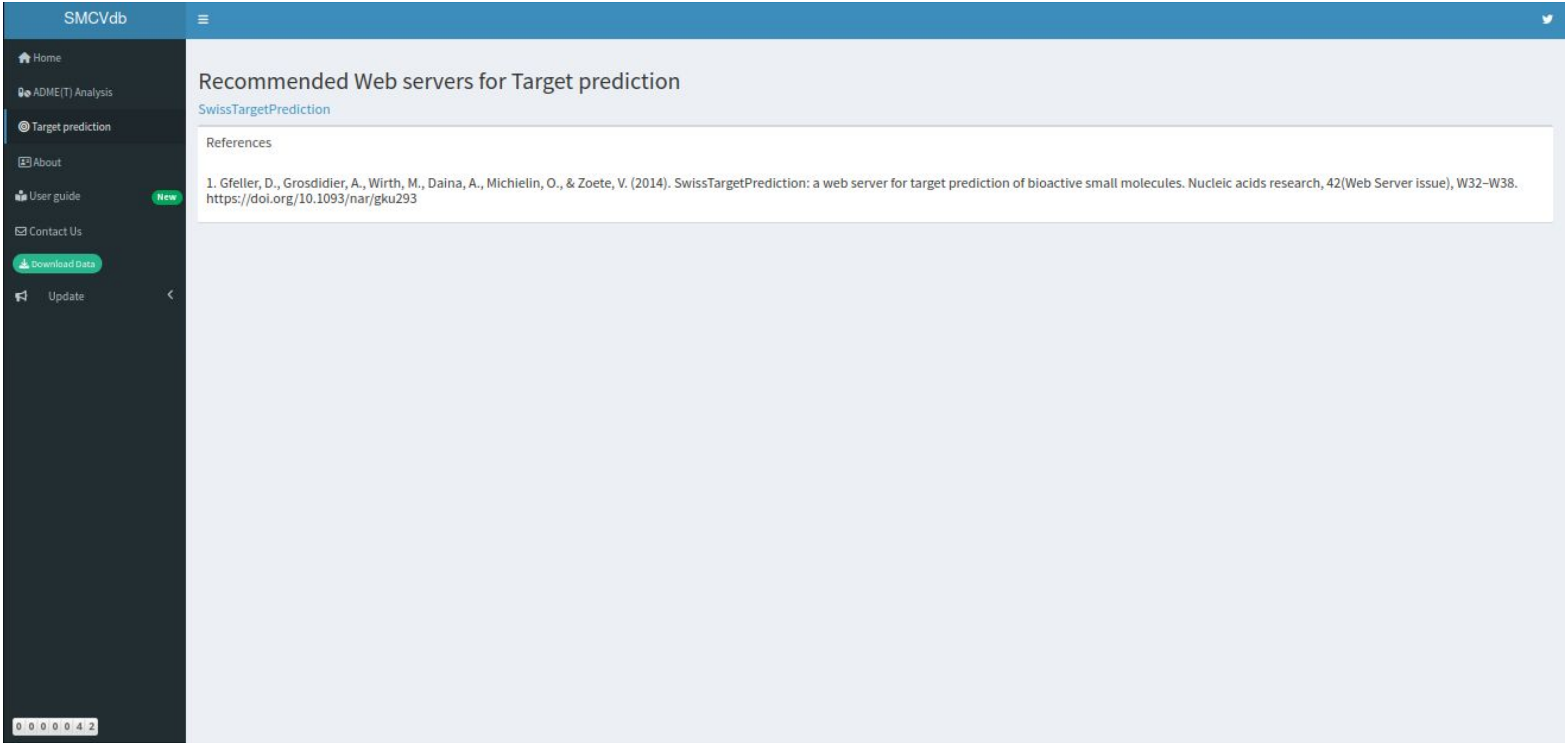

Case 9: Target prediction for a specific compound

Tutorial main

Step 4 : Paste the SMILES notation and click RUN

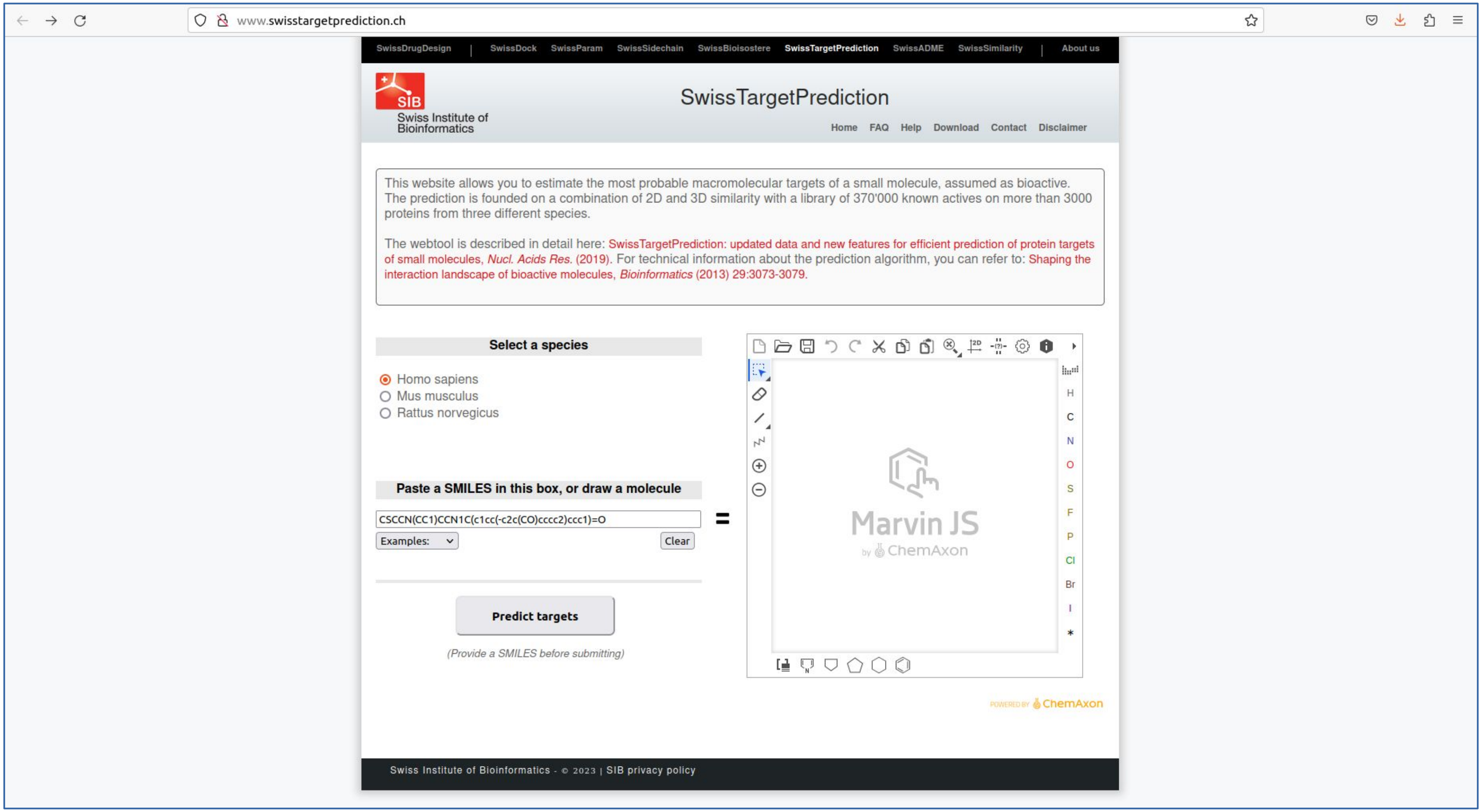

Source : http://www.swisstargetprediction.ch/

Case 9: Target prediction for a specific compound

Step 5 : Result page will appear

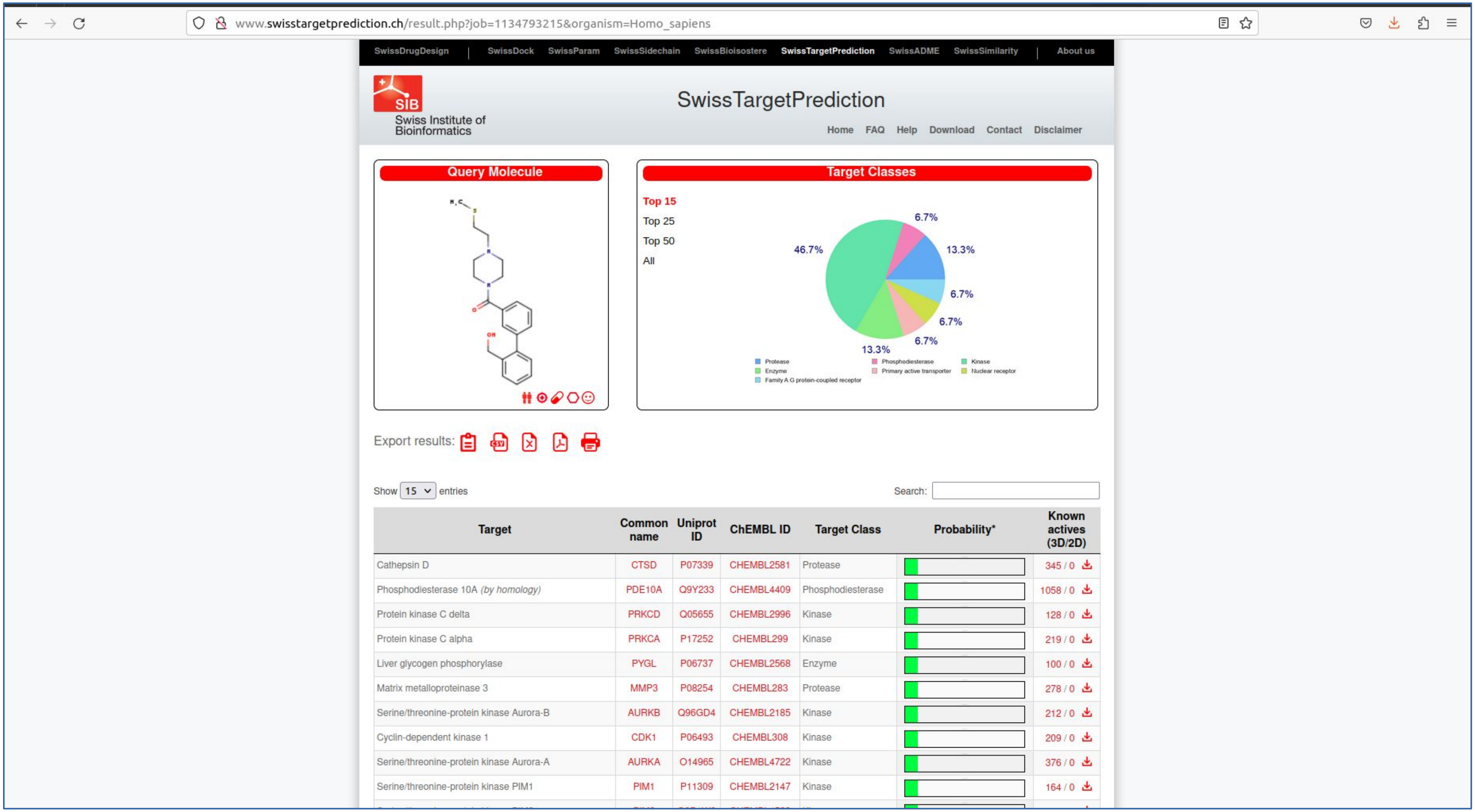

Target	Common name	Uniprot ID	ChEMBL ID	Target Class	Probability*	Known actives (3D/2D)
Cathepsin D	CTSD	P07339	CHEMBL2581	Protease		345 / 0
Phosphodiesterase 10A *(by homology)*	PDE10A	Q9Y233	CHEMBL4409	Phosphodiesterase		1058 / 0
Protein kinase C delta	PRKCD	Q05655	CHEMBL2996	Kinase		128 / 0
Protein kinase C alpha	PRKCA	P17252	CHEMBL299	Kinase		219 / 0
Liver glycogen phosphorylase	PYGL	P06737	CHEMBL2568	Enzyme		100 / 0
Matrix metalloproteinase 3	MMP3	P08254	CHEMBL283	Protease		278 / 0
Serine/threonine-protein kinase Aurora-B	AURKB	Q96GD4	CHEMBL2185	Kinase		212 / 0
Cyclin-dependent kinase 1	CDK1	P06493	CHEMBL308	Kinase		209 / 0
Serine/threonine-protein kinase Aurora-A	AURKA	O14965	CHEMBL4722	Kinase		376 / 0
Serine/threonine-protein kinase PIM1	PIM1	P11309	CHEMBL2147	Kinase		164 / 0

Source : http://www.swisstargetprediction.ch/

Case 10: User support

Tutorial main

Option 1 : Use contact us option

Step 1 : Click Contact Us link

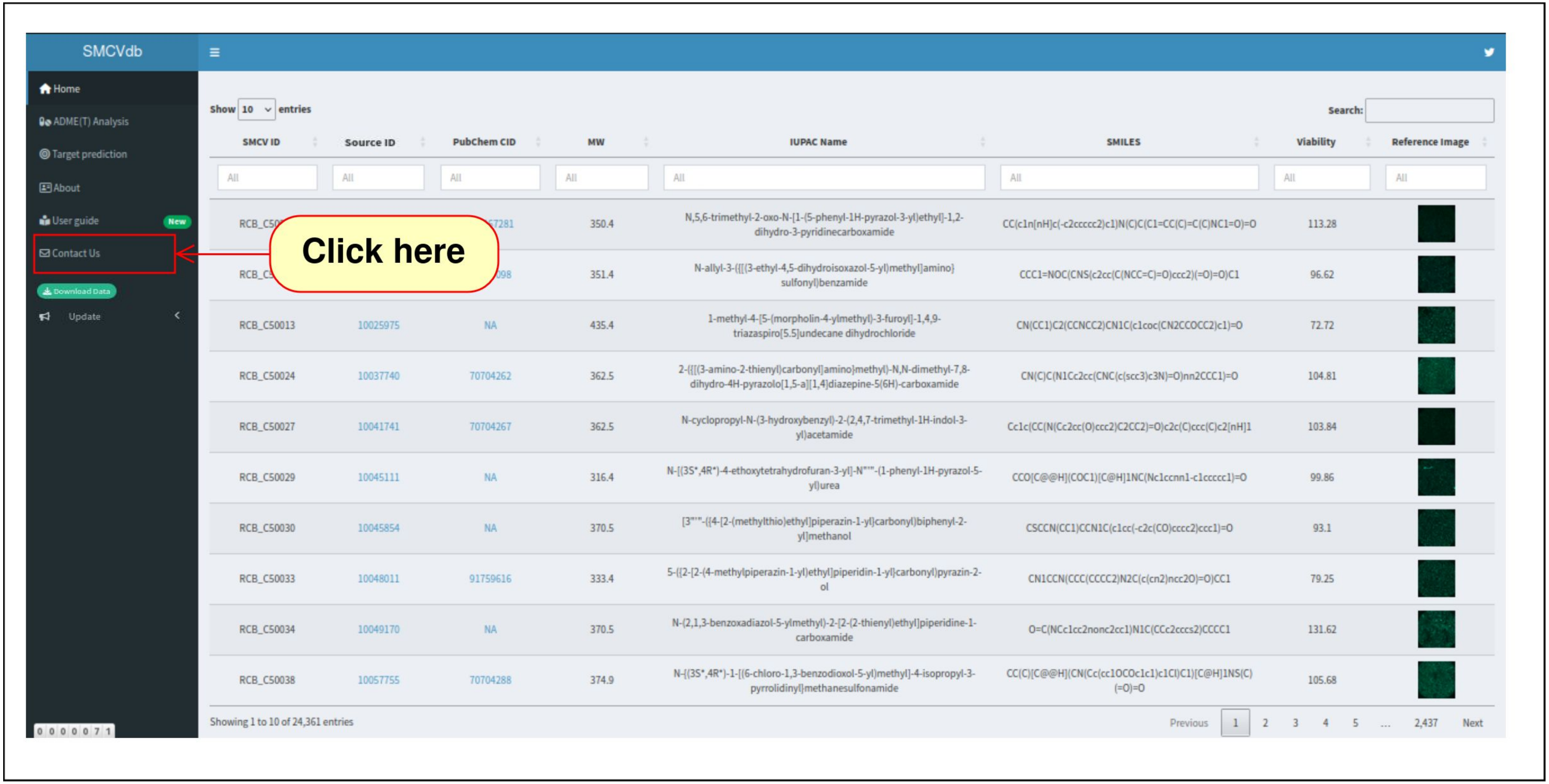

Case 10: User support

Tutorial main

Option 1 : Use contact us option

Step 2 : Submit the form with details

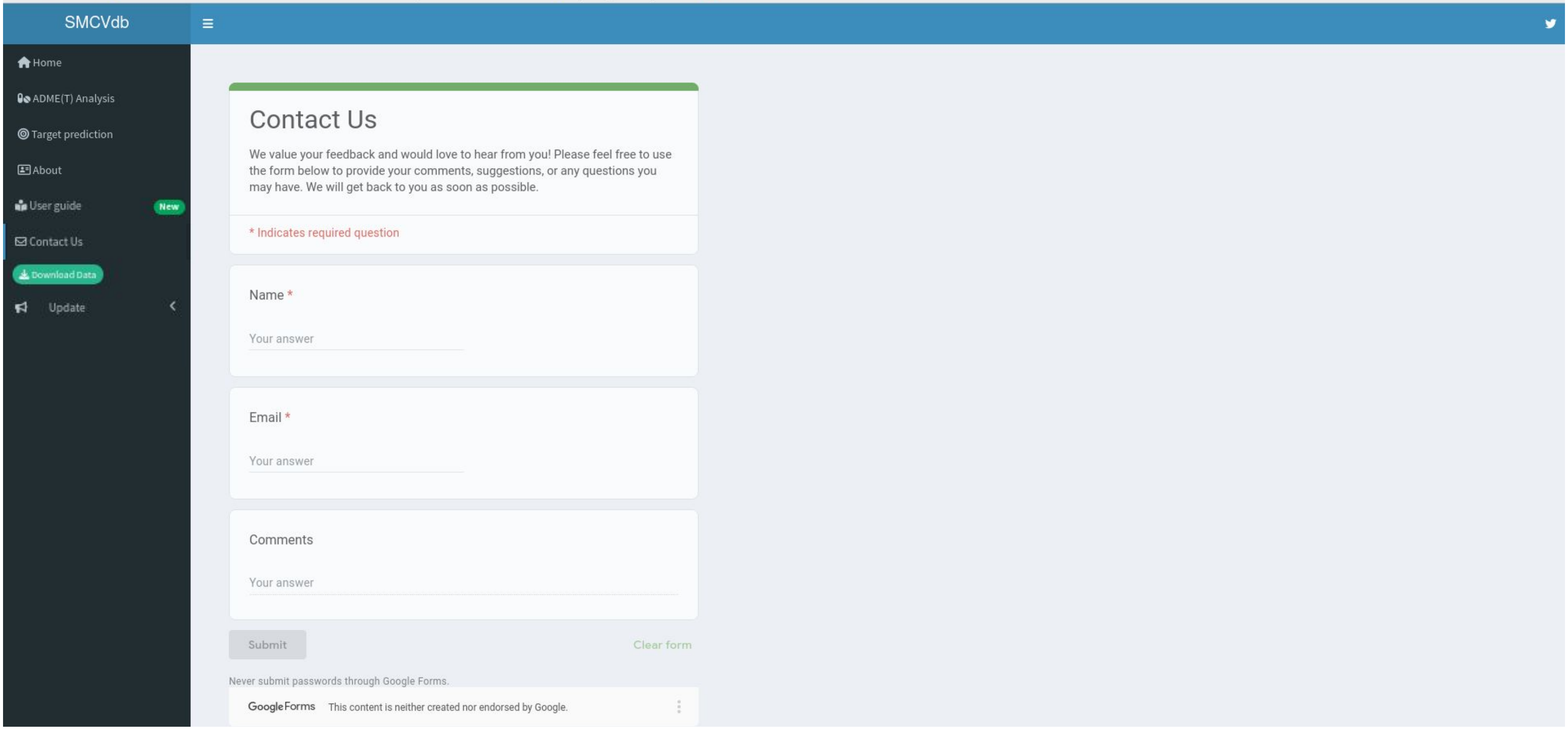

Case 10: User support

Tutorial main

Option 2 : Follow us on social media page

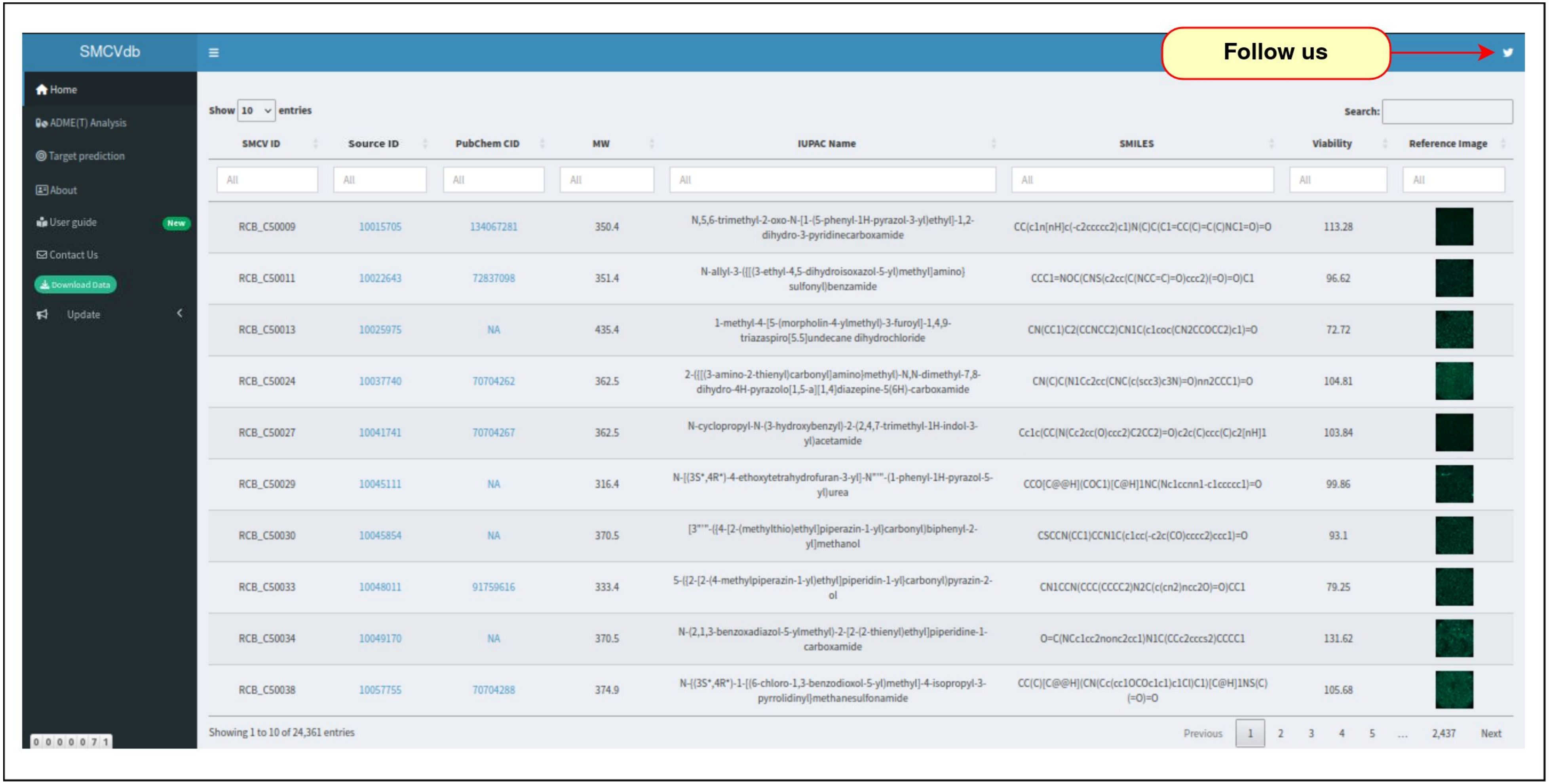

Supplement: baae100_Supp [file baae100_supp.zip › suppl_data/tutorial_19082024.updated.pdf]
